# Quality, Non-clinical and Clinical Considerations for Biosimilar Monoclonal Antibody Development: EU, WHO, USA, Canada, and BRICS-TM Regulatory Guidelines

**DOI:** 10.3389/fphar.2018.01079

**Published:** 2018-09-28

**Authors:** Hasumati Rahalkar, Hacer Coskun Cetintas, Sam Salek

**Affiliations:** ^1^School of Life and Medical Sciences, University of Hertfordshire, Hatfield, United Kingdom; ^2^Department of Regulatory Sciences, Metina PharmConsulting Pvt Ltd, Mumbai, India; ^3^Department of Marketing Authorization of Medicines, Turkish Medicines and Medical Devices Agency, Ankara, Turkey

**Keywords:** monoclonal antibody, biosimilar, regulatory, comparability, quality, manufacturing, non-clinical, emerging markets

## Abstract

**Objective:** The aim was to critically evaluate well-established regulatory agencies mAb biosimilar guidelines for development and marketing authorization about quality, efficacy and safety and compare to BRICS-TM regulations to identify challenges.

**Materials and Methods:** The current valid guidelines of EMA, WHO, USFDA, BGTD/HC, ICH, and BRICS-TM were obtained from official websites and comparative qualitative review was performed.

**Results:** The review revealed that Health Canada uses mAb specific guidelines from EMA or USFDA when necessary. The BRICS agencies (except Russia) have incorporated some or most of the WHO SBP TRS and related annexes in similar national biotechnological/biological guidelines; however, gaps or insufficient information have been identified. The Russian Federation has issued general product registration guideline/s with very brief information about mAbs. The TMMDA (Turkey) has published an updated biosimilar guideline which parallels those of the EMA and the ones from WHO; however, no mAb specific guidelines are published. COFEPRIS (Mexico) has published a biotechnological/biological product registration guideline with no information about mAb. The SAHPRA biosimilar guideline has an annex on mAbs which focuses on non-clinical and clinical aspects.

The comparative evaluation of BRICS-TM agencies indicates a gap pertaining to clarification for physico-chemical characterization, manufacturing process, overages and compatibility requirements between biological substances and excipients specifically on mAbs. *In vitro* assay requirements seem quite aligned with those of WHO, whereas *in vivo* studies mostly have disparity in terms of necessity, type of studies as well as design and criteria. Clinical safety and efficacy studies are indicated in emerging regulatory agencies, however detailed information pertaining to design, size of populations, requirements for primary and secondary endpoints, clarity and evaluation criteria differ. In general, BRICS-TM agencies allow extrapolation of indications provided that pre-defined conditions are met. Interchangeability, switching and substitution of biosimilars are not defined in most of BRIC-TM guidelines whereas South Africa, by law, allows neither interchangeability nor substitution. Pediatric research remains questionable across BRICS-TM.

**Conclusions:** EMA, USFDA guidelines are broadly aligned with WHO and in addition, they also contain specific requirements pertaining to their own region. BRICS-TM has considerably less defined mAb specific biosimilar development and comparability parameters in their published guidelines.

## Introduction

Biosimilars are biotherapeutic products with identical quality and similar safety and efficacy profiles as the reference biological product. It is essential that the standard of evidence supporting the decisions to grant marketing authorization for biosimilars be sufficient to ensure that the products meet acceptable levels of quality, safety and efficacy for public health purposes.

Biosimilars cover specific products such as low molecular weight heparins, insulin, human follicular stimulating hormones, interferon's, erythropoietin, granulocyte stimulating factor, monoclonal antibodies. Amongst all, the class of monoclonal antibody (mAbs) products is getting higher importance due to its use in chronic diseases like rheumatoid arthritis, cancers, ulcerative colitis, Crohn's disease etc. Monoclonal antibodies are complex biotherapeutic proteins known as immunoglobulins (Ig) and are derived from different technologies such as hybridoma, recombinant DNA (rDNA), B lymphocyte immortalization etc. To develop clinically equivalent mAb to prove comparability with reference biological product raises multiple challenges starting from reference product selection, characterization (physico-chemical, manufacturing process, *in vitro/vivo* assays), clinical safety and efficacy.

Although the European Medicines Agency (EMA), the United States Food and Drug Administration (USFDA) and the World Health Organization (WHO) have issued specific guidelines with questions and answer documents clarifying doubts pertaining to development, many other agencies are yet to develop mAb specific regulatory guidance. A few emerging markets such as Brazil, Russia and Mexico have very brief or unclear mAb/biosimilar guidelines. Indian and Chinese guidelines are in line with those of the WHO, whereas those of South Africa and Turkey are mostly aligned with the EMA guidelines with minor differences. Supplementary Table 1 indicates list of authorized mAb biosimilars in each market between 2013 and 2018. The information has been obtained by online search on official websites and extracted from related published articles.

In this review, we present quality, non-clinical and clinical requirements for the development of biosimilar mAbs and for their licensing by the EMA (overview of EMA guidelines related to the development and approval of biosimilars) (Schiestl et al., 2017), the WHO, USFDA, BGTD/HC, ANVISA/Brazil, Russian Federation/Russia, CDSCO/India, CFDA/China, SAHPRA/South Africa, TMMDA/Turkey, COFEPRIS/ Mexico. We have referenced and interpreted general biosimilar guidelines where guidelines specific to mAbs are unavailable. To get an overall understanding, we have also reviewed and cross-referred ICH guidelines (ICH, 1995a,b, 1997, 1999a,b,c, 2004a,b, 2012). The biosimilar and reference product are defined using varied terminologies by each agency. For ease of understanding, we have followed agency specific terminologies for review and comparison, throughout this article.

## Materials and methods

The current and valid English-language guidelines including published questions and answers such as the EMA guidelines pertaining to biosimilar medicinal product and mAbs, technical report series (TRS) and pertinent annexes of the WHO, specifically for mAbs, guidance for industry from USFDA, the guidance document and the Fact sheet issued by BGTD/HC, MCC (currently known as SAHPRA) /South Africa guidance document and guidelines on similar biologics from India which were obtained from the official websites of the respective regulatory agencies.

Non-English language guidelines such as resolution RDC n° 55/2010 published by ANVISA, the technical guideline for R&D and evaluation of biosimilars, issued by the Center of drug evaluation (CDE) China on 28th February 2015, the draft guideline on biosimilar medicinal product TMMDA, the official Mexican standard NOM-257-SSA1-2014 for biotechnological medications, were translated into English by a professional agency and/or a translated version was obtained from local resources for further review.

Apart from the national guidelines, the quality considerations highlighted in this article are based on ICH Q5A to Q5E- quality of biotechnological products guidelines, ICH Q6B for specifications and ICH Q11 for the development and manufacture of drug substances (chemical entities and biotechnological/biological entities). The reviewed ICH guidelines were those published during November 1995 to November 2004 whereas national guidelines were issued from 2002 until February 2018. Refer to Supplementary Table 2 presenting country, agency and guidelines referred for detailed review.

The main aim of the research was to evaluate and identify gaps in regulations and marketing authorization practices followed in emerging markets, hence well-established agencies i.e. EMA, WHO, USFDA and BGTD were selected as reference agencies. The criteria to select reference agencies are as follows: **EMA** is considered as a pioneer in biosimilar regulation development and the first agency to come up with detailed regulations for biosimilars. Apart from that, many emerging agencies are following EMA's published biosimilar guidelines as reference documents in the absence of detailed guidelines published by their own agency. When it comes to emerging agencies, the **WHO** regulatory framework is taken as the benchmark for developing national regulations. Hence WHO is considered a reference agency. The **USFDA** is considered as an advanced regulatory agency and believed to be more transparent and vigorous in terms of regulatory expectations. **BGTD Canada** is an ICH member country and co-chair to International Pharmaceutical Regulators Forum (IPRF). It also works in close association with emerging agencies i.e. Brazil, Russia and Mexico. At the moment, **PMDA Japan** is excluded from the research since most of the emerging agencies follow EMA, WHO, USFDA or BGTD for biosimilars. The **TGA Australia** is excluded since the regulations are fully aligned with those of the EMA and no differences in quality, nonclinical and clinical requirements can be observed.

The emerging agencies i.e. Brazil, Russia, India, China, South Africa, Turkey, and Mexico are selected due to their growing emerging economies, increased patient populations, demand for biosimilars and evolving regulatory agencies.

The data pertaining to biosimilarity principles, comparability exercise, selection of reference product, physico-chemical characterization, manufacturing process and specification determinations, *in vitro* and *in vivo* studies, pharmacokinetic (PK) and pharmacodynamic (PD) studies, immunogenicity assessment, PK and PD studies in humans, comparative clinical trials, clinical safety studies, extrapolation to other indications, pharmacovigilance and interchangeability were extracted from the aforementioned guidelines. Subsequently, the data were qualitatively compared, and visible similarities and/or differences were identified for emerging markets.

## Results

**Figure 1:**
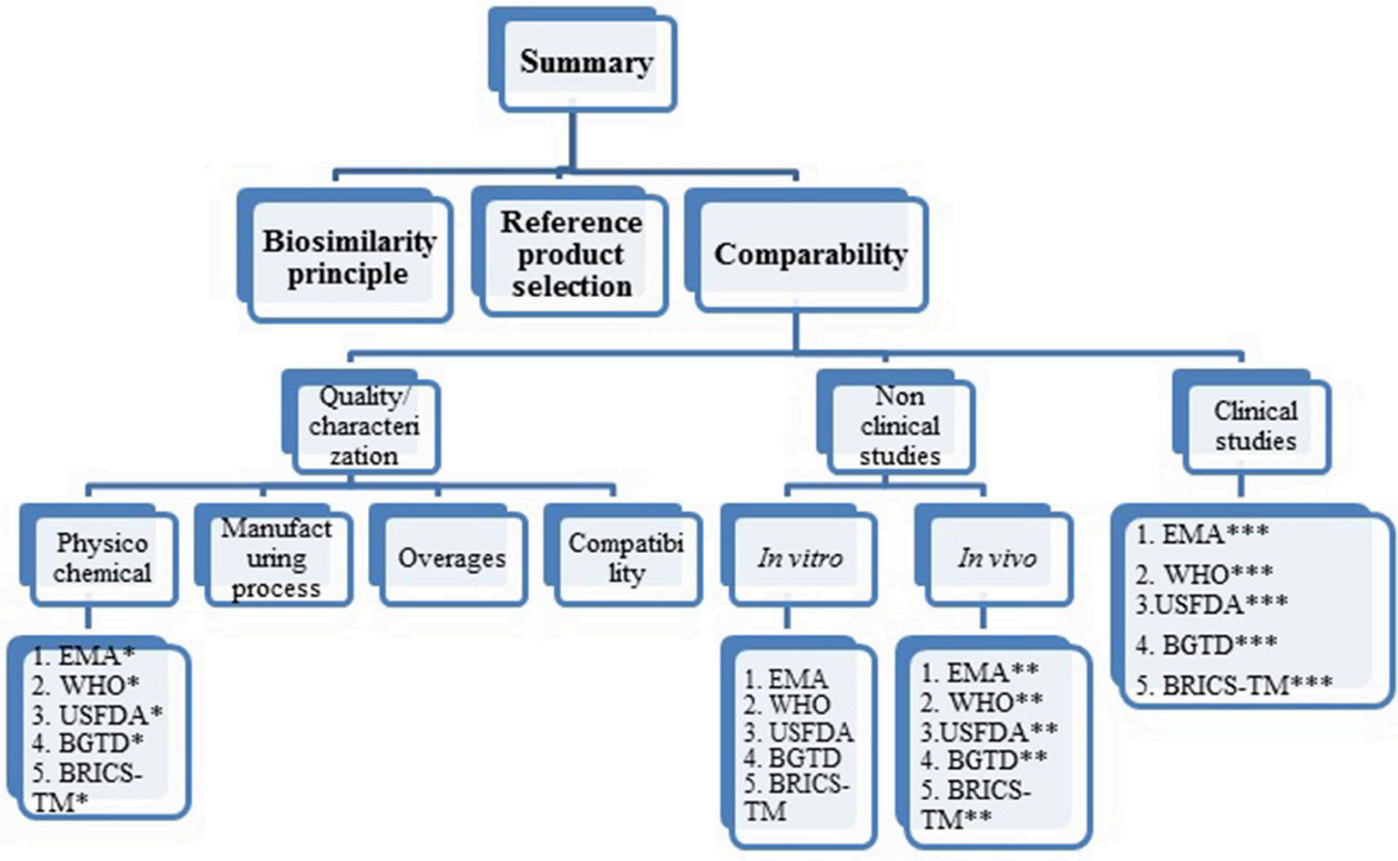
Structure of data presentation in result section. *Under each agency the psychochemical requirements covered are mAb structure, immunological properties, biological activity purity impurity and contaminants cell lines quantity, specifications. **Under each agency, the *in vivo* (non-clinical) studies requirements covered are pharmacokinetics, pharmacodynamics, immunogenicity, safety pharmacology, toxicology, carcinogenicity, local tolerance. ***Under each agency the clinical requirements covered are pharmacokinetics, clinical efficacy, clinical safety, extrapolation to other indications, pharmacovigilance, risk management plan.

The summary of this review research is presented as biosimilarity principles, reference product selection and comparability exercise. The comparability criteria are further specified into quality/characterization (physicochemical characterization, manufacturing process, overages and compatibility), non-clinical studies (*in vitro* and *in vivo studies*), clinical studies (PK and PD studies, clinical efficacy and clinical safety studies, extrapolation to other indications, pharmacovigilance and risk management plan). Each criterion is further divided, based on agency in sequential order as EMA, WHO, USFDA, BGTD/HC and BRICS-TM agencies. For ease of understanding, refer to Figure 1 pertaining to structure of data presentation in the results section.

### Biosimilarity principles

The biosimilars are therapeutically similar to an originator biological product and proving these attributes involves comparative analytical, non-clinical and clinical testing with the reference product selected (Olech, 2016). The objective of the development plan of such products is to prove similarity in terms of quality and efficacy to the reference biological product. With regards to safety, better safety profile is allowed; they can also expect to have lower impurity profile than the originator.

The biosimilarity principles encompass terminology, development approach, basis of biosimilarity, demonstration of biosimilarity with the chosen reference product, simplified approach and regulatory framework across advanced regulatory agencies and national regulatory authorities of BRICS-TM. Different terminologies such as similar biological medicinal product, biosimilar, similar biological product, biosimilar biological drug are being used to define similar versions of reference biological/ biotechnological products. The stepwise development approach for characterization, *in vitro* and *in vivo* non-clinical studies and clinical studies is uniform across well-established regulatory agencies and a totality-of-the-evidence approach for assessment of biosimilarity is followed. However, approach to biosimilarity is not clear in some of the agencies of emerging markets.

In general, according to a step-wise approach, the biosimilarity of a proposed biological product should be demonstrated by the totality of the evidence, whereas minor quality differences may be justified by pre-clinical or clinical studies. Additionally, WHO indicates the need for safety data for differences that are not explained. In addition, companies may use various tools such as technical advice or consultation to receive advice on scientific matters by discussing biosimilar development with advanced regulatory agencies such as USFDA and EMA by arranging formal meeting (CBER, 2018). When it comes to biosimilarity demonstration, Brazil, Russia and Mexico stresses more comparative clinical studies or no defined recommendations in published guidelines.

Depending upon the demonstration of biosimilarity, the application can be filed as stand-alone or an abbreviated filing with ANVISA, Brazil. Simplified approaches (in terms of smaller clinical trial/s if bioassay is known to be clinically relevant or the number of patients may vary depending upon the endpoints) is defined by the EMA, WHO and USFDA with prior regulatory opinion; however, such transparency is yet to be defined in detail by other BRICS-TM agencies except TMMDA and SAHPRA (Anvisa, 2010; CFDA, 2014; Cofepris, 2014; MCC, 2014; Russian-federation, 2014; CBER, 2015; EMA, 2015; CDSCO, 2016; General-Directorate-for-Pharmaceuticals-and-Pharmacy, 2017; WHO, 2017; WHO-TRS, 2017).

### Reference product selection

The reference biological product selected for comparability studies must be licensed with full dossier including quality, safety and efficacy. For most agencies, it must be sourced from the same territory where biosimilar authorization is requested. Supplementary Tables 3, 4 refer to reference product characteristics according to advanced agencies i.e. EMA, USFDA, BGTD/HC and BRICS-TM agencies, respectively. The WHO leaves the decision to the NRA to define criteria for the reference product selection; this is evidence based on factors for selection of the reference biologic determined by CDSCO, India (Rushvi et al., 2016).

The (EMA, 2014b) guidelines state acceptance of non-EEA authorized comparator for certain clinical studies and *in vivo* studies during development of Quality targeted product profile (QTPP) of the proposed biosimilar product, subject to such comparator being authorized by regulatory authorities having similar scientific and regulatory standards to the EMA. The bridging data is expected in terms of quality, non-clinical or clinical side-by-side comparability between the reference product, non-EEA authorized comparator product and proposed biosimilar product. In the EU, the data generated from another country's reference product can be used as supporting data for biosimilarity assessment. The EMA and USFDA expect that when there is usage of a third country reference product, in addition to bridging data generation, the pathway is to be discussed in advance, whereas, a similar approach is yet to be defined with emerging market regulatory agencies (Anvisa, 2010; CFDA, 2014; Cofepris, 2014; MCC, 2014; Russian-federation, 2014; CDSCO, 2016).

The reference product must be identified in terms of brand name, pharmaceutical form, strength, number of batches, lot number and age of batches. It is expected that the originator product will be used and multiple batches with different shelf lives remaining throughout the development and comparability studies will be tested. As for WHO, if the reference biological product is manufactured at different locations but authorized under a single license for global distribution, then it will be considered as the same reference product. In such scenarios, the reference product manufactured in different sites can be used as RBP for biosimilar development (Health-Canada/BGTD, 2012, 2016; WHO-TRS, 2013; CBER, 2015; EMA, 2015; General-Directorate-for-Pharmaceuticals-and-Pharmacy, 2017).

Biosimilar products are not allowed to be used as reference products across all agencies, except COFEPRIS (Mexico). It is observed that only COFEPRIS accepts a biosimilar product as a reference product in case of **non-availability** of a reference product (Cofepris, 2014).

### Comparability exercise

The comparability exercise is a crucial element to prove similarity of a proposed similar biological product. It involves side by side comparison of sequential comparative studies against a reference product starting from characterization (e.g., physico-chemical characterization, manufacturing process, compatibility, overages) of the active substance to the final product, followed by non-clinical and clinical studies. In general, each agency expects that significant differences observed in previous steps are to be explained in subsequent studies. The justified variations in excipients, cell lines, manufacturing processes, container closures, test parameters, impurities, storage conditions, shelf life and lower immunogenicity may fall within the scope of biosimilarity.

Differences in safety parameters, such as reduced toxicity or number and type of adverse reactions, may not prevent the establishment of biosimilarity. However, efficacy differences, e.g., altered glycosylation profile would most likely be considered outside the bounds of biosimilarity principles.

A further aspect related to extrapolation of indications is the shift from acceptance to conditional acceptance (Supplementary Table 5). It is clearly visible that interchangeability decision falls within the scope of each member states of the EU (Minghetti et al., 2012), whereas for Canada, the decision of is out of scope of regulators but in hands of province/territory. The USFDA allows interchangeability subject to additional studies, whereas this often remains unspecified in the less mature regulatory agencies. Pediatric research or extrapolation of efficacy in the pediatric population is indicated under PREA by US authority whereas the same is at a very basic stage in emerging markets (Supplementary Table 5; EMA, 2007; Anvisa, 2010; WHO-TRS, 2013; CFDA, 2014; Cofepris, 2014; EMA, 2015; MCC, 2014; Russian-federation, 2014; CBER, 2015a,b, 2017; EMA, 2015; CDSCO, 2016; General-Directorate-for-Pharmaceuticals-and-Pharmacy, 2017; Health-Canada/BGTD, 2017).

#### Quality/characterization

##### Physico-chemical characterization

A typical antibody is made up of four polypeptide chains wherein each chain consists of one heavy chain and one light chain, connected via disulfide bridges or bonds and has amino terminal variable region/s. The number of amino acids at amino terminal varies for different antibodies. The complementarity determining region (CDR) is the most variable part of the variable region and it comes together at an amino terminal to form an antigen binding site. The class and sub-classes of monoclonal antibodies are defined based on isotypic, allotypic and idiotypic determinants.

The Y-shaped antibody is made up of one constant region (Fc) and two variable regions (Fab) with antigen binding sites. The Fc region exhibits limited variability and is made up of two light chains and five heavy chains, with linkage of carbohydrate group at the heavy chain (Wiley-publication, 1998).

**Figure 2:**
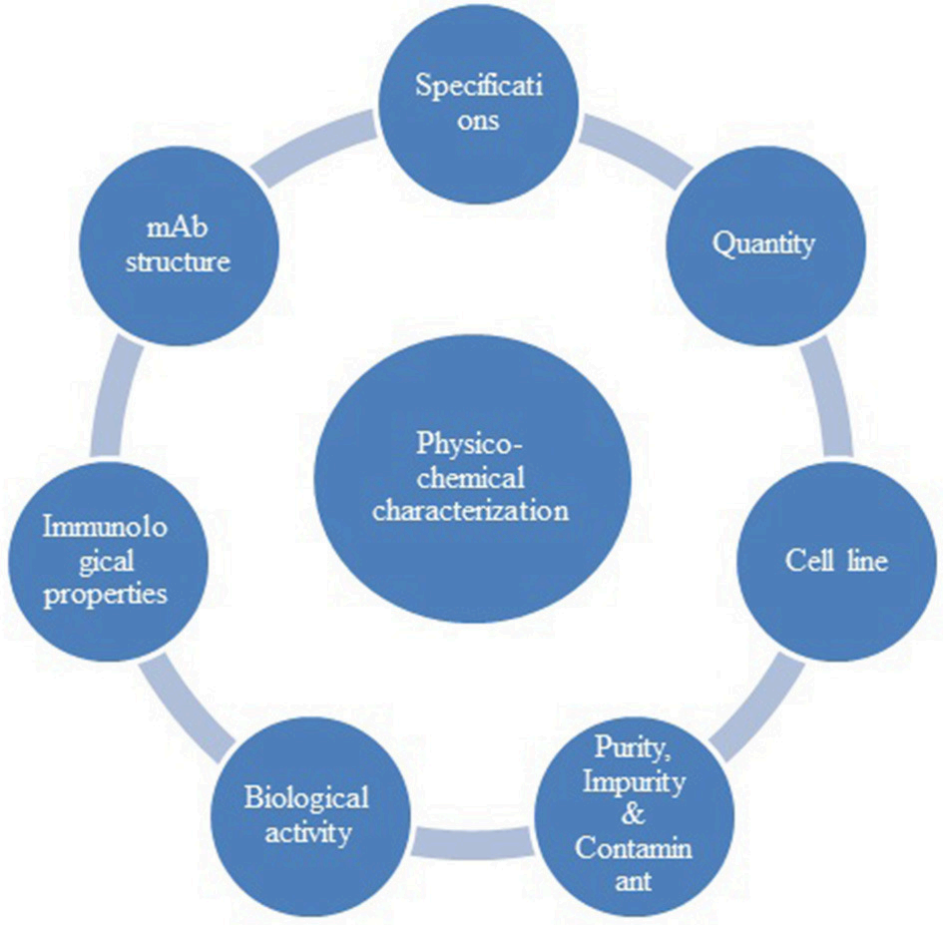
Monoclonal antibodies-Physiochemical characterization parameters.

The primary step of the comparability exercise starts with characterization of a reference biological product to establish the Quality targeted product profile (QTPP) of the proposed biosimilar. While performing characterization of a reference product containing an ingredient i.e. albumin, the extraction of albumin is expected by appropriate extraction methods so that the structure of the mAb is not modified. The physicochemical requirement for a mAb is indicated in Figure 2. The information provided herewith is based on EMA and WHO guidelines specific to monoclonal antibodies. BRICS-TM agencies, except SAHPRA, have not published a specific mAb guideline.

##### EMA

*mAb structure:* As part of physico-chemical characterization of mAbs, the EMA has recommended performing primary and higher order structure identification covering class and sub-classing determinations, the type of chain (kappa and/or lambda) involved and detailed characterization of primary structure. Guidelines further specify requirements pertaining to amino acid sequencing as well as conformation of amino and carboxy terminal at Fab- and Fc- region respectively. The intra and inter disulfide bridges to be determined along with their integrity and mismatches with respect to the reference medicinal products. The free sulfhydryl groups are to be identified as well. The carbohydrate content, its structure and oligosaccharide pattern are to be identified and confirmed. The glycosylation site on the Fc- region is required to be analyzed with its occupancy. The presence or absence of glycosylation site is to be confirmed and characterization to be done if confirmed positive. Apart from the above, the glycan structure is to be characterized with mannosylation, galactosylation, fucosylation, and sialylation with distribution of main glycan structure.

Guidelines on similar biological medicinal products (EMA, 2014b, 2015, 2016) define requirements pertaining to all biotechnological/biological products including monoclonal antibodies. The physico-chemical characterization section of this guideline states requirement for description for structural elements such as active sites, receptors, ligand binding sites and features for signal transduction. Additional characterization with respect to *in vivo* disposition of active substance while administering product and interactions between active substance and excipients would be necessary.

*Immunological properties*: The antigen binding fragment (Fab) region of a mAb is critical for its immunological properties. EMA recommends analyzing antigen binding assay at defined regions including affinity, avidity and immunoreactivity, as feasible to be performed. In addition to binding assay, cytotoxicity evaluation at unintended target issue and cross-reactivity need to be determined. The epitope (the part of antigen which attaches to an antibody) needs to be characterized, biochemically identified and determined including the base molecules. The identification of complementarity determining regions is essential or needs to be justified. It is further stated that evaluation of binding and activation and/or effector functions should be evaluated even though the proposed biological activity does not demand such function. The above immunological studies are expected to be performed against the reference biological product and must be comparative in nature.

*Biological activity*: The biological activity is generally defined as the ability of a product to give biological results/effects. EMA expects appropriate *in vitro* assay(s) for assessment of biological activity. The *in vivo* assays if performed need detailed justification. The mechanism of action including its importance and consequences of product effector functions with respect to safety and efficacy of product calls for discussions. Such discussions should be based on a detailed analysis of antibody-dependent cell-mediated cytotoxicity (ADCC), cytotoxic properties (e.g., apoptosis), complement binding and activation ability, other effector functions i.e., Fc- gamma receptor binding activity and neonatal Fc- receptor (FcRn) binding activity. The above biological studies are expected to be performed against the reference biological product and must be comparative in nature.

*Purity, Impurity and Contaminants*: mAbs can express heterogeneity due to multiple factors such as C-terminal lysine processing, N- terminal pyroglutamate, deamidation, oxidation, isomerization, fragmentation, disulfide bond mismatch, N-linked oligosaccharide, glycation. This results in different molecular entities for mAbs. To identify purity or impurity profiles of mAbs, the orthogonal methods which include physico-chemical property determinations need to be performed.

The formation of aggregates, sub visible and visible particulates needs investigation and close monitoring during batch release and stability studies. Multimers and aggregates need to be characterized appropriately.

Process related impurities such as host cell DNA, cell culture residues, downstream processing residues demand identifications as well as qualitative and/or quantitative evaluation.

Contaminants (outside the scope of the manufacturing process) are expected to be controlled or restricted. Appropriate additional testing is required to be done, if pro-inflammatory contaminants are suspected.

*Cell lines:* Sufficient monoclonal cell line information is expected, but detailed specific procedures prior to the isolation of the monoclonal cell line i.e. cell fusion, viral transformation, gene library of phage display screening, application of *in silico, in vitro* or *in vivo* technologies are not required to be described in great detail. Origin and characteristics of parental cells need to be documented and immortalization approach to be defined.

*Quantity*: The quantity of finished product should be determined based on appropriate physicochemical and/or immunochemical assays. The same can be determined based on biological assays subject to demonstration of correlation between quantity and biological activity.

*Specifications*: Specification is determined based on batch data, manufacturing experience, characteristics of the product, other controls used in the process etc. The ICH Q6B should be followed for drug substance and drug product for test selection. In general, more than one specific identity test, purity, impurities, potency and biological activity test to be included. The glycosylation test should be performed for the products where posttranslational modification could occur. Other general tests as applicable to formulations need to be covered e.g., solubility, extractable volume, bacterial endotoxin.

The acceptance criteria need to be defined based on lots used in studies i.e. manufacturing consistency, non-clinical and clinical studies, stability studies and other relevant development data. The analytical method validation is to be submitted as part of the marketing authorization application dossier. Compendial reference methods are expected to be used from European pharmacopeia (Ph Eur) and from WHO.

During pre-formulation studies, the stability of active substances needs to be proved by establishing degradation pathways whereas for formulation, experimental data with different quantities of suitable excipients is expected. If the product is lyophilized, then the usage of lyoprotectant must be determined for process optimization through in-process stability study. Real time, real condition stability studies are required as part of routine stability studies in line with ICH Q5C (EMA, 2000, 2016).

##### WHO

*mAb structure:* Supplementary Table 6 presents differences between EMA and WHO for mAb physicochemical characterization requirements. Although WHO has defined primary and higher order structure characterization, specific mandates pertaining to class and sub-class determination and kappa and/or lambda chain confirmation remain unspecified, as it is the case with the EMA. The carbohydrate structure, glycosylation pattern and glycan evaluation requirement are the same as those of the EMA.

WHO does not refer to structural elements, *in vivo* disposition, interactions, amino acid sequencing with variability at N- and C- terminal, disulfide bridges and free sulfhydryl group requirements. The rest of the parameters of characterization are aligned with those of the EMA.

*Immunological properties*: The expectations pertaining to binding assays are spelled out in guideline without further detail.

*Biological activity*: It is expected that appropriate assays will be performed; however, there is no clarity with respect to *in vitro* or *in vivo*. Further it is stated that ADCC binding ability to Fc- gamma receptor binding activity, neonatal Fc receptor (FcRn), complement (C1q) binding, Fc and Fab related functions are to be performed. It is unclear whether such test is required; in the case the ADCC-related mechanism of action does not impact safety and efficacy.

*Purity, Impurity and Contaminants:* Investigations, identifications and quantifications of structural heterogeneity are expected.

Impurity profiling as well as process related impurities are required to be considered, however use of orthogonal methods is not defined, either for purity or impurity tests. The status of non-clarity prevails for multimers, aggregates, particulates, and contaminants.

*Cell lines:* The SBP manufacturer/applicant can use different monoclonal cell lines than the reference biological product (RBP) to produce a biosimilar mAb, when the structure of the molecule and its clinical profile remain unchanged; but in-depth requirement is not specified. However, it is recommended to use the same monoclonal cell line as RBP.

*Quantity*: The quantity of biosimilar product in final presentation is determined based on biological activity and expression system.

*Specifications*: It is expected that specifications will be determined based on the manufacturer's experience with similar biologic product (SBP) and experimental results with SBP and RBP. The tests are to be performed as per pharmacopeia monograph plus additional test as appropriate. The acceptance criteria are to be decided based on a sufficient number of lots and should not be wider than the variability range of the RBP during entire shelf life.

The analytical methods for characterization should be scientifically sound and qualified but it is not necessary that they be validated, whereas for lot release validation is expected. The reference materials and standards are expected to be sourced from the WHO.

Real time and stability under real conditions for the SBP is expected, whereas experimental stability data and in process stability data are not defined. It is expected to have comparative head-to-head accelerated stability studies between SBP and RBP and non-comparable stress studies. Apart from this drug product and drug substance, stability studies are expected in intended and in the representative container closure system (WHO-TRS, 2013, 2017; WHO, 2017).

##### USFDA

*mAb structure:* The physico-chemical characterization requirements are specified in guidance documents pertaining to biotechnological/biological products. The PEGylation characterization is unique to USFDA requirement. Appropriate physico-chemical methods i.e. sodium dodecyl sulfate polyacrylamide (SDS-PAGE), Isoelectric focusing (IEF), High-performance liquid chromatography (HPLC), mass spectrometry (MS) are required to be used. Binding assays to be performed and epitope should be defined biochemically; the quantification of antibody binding activity needs to be performed by combination of tests such as affinity, avidity and immunoreactivity.

*Immunological properties:* The requirement is in line with WHO immunological properties.

*Biological activity: in vitro* and *in vivo* assays are required without further recommendation on product effector functions.

*Purity, Impurity and Contaminants:* The risk of impurities and contaminants are additional for biological products due to usage of living systems for manufacturing (Christl et al., 2017). It is expected that structural heterogeneity and aggregates will be characterized. The purity and impurity tests are to be performed using orthogonal methods whereas there is need to characterize for known and potential impurities. The contaminants expectation remains in line with those of the EMA.

*Cell lines:* It is recommended to minimize differences between reference product's expression construct and the one proposed for the biosimilar product: justification for differences is to be provided.

*Quantity:* Potency must be defined based on assay(s).

*Specification:* Accelerated and stress stability studies under multiple stress conditions such as high temperature, freeze thaw, light exposure and agitation are required for appropriate physico-chemical and functional comparison of the stability profile of the proposed product against that of the reference product. Sufficient real time and real condition data of the proposed product is to be provided in support of the shelf life (CBER, 2015a,b).

##### BGTD/Health Canada (HC)

*mAb structure:* BGTD/HC has not published a guidance document exclusive to mAbs. However, it has advised to reference ICH Q5E pertaining to comparability of biotechnological/biological products subject to changes in their manufacturing process. In line with ICH Q5E, higher order (secondary, tertiary and quaternary) structures for mAb are to be determined for proving a suitable comparability exercise for the proposed biosimilar. About amino acid sequencing, disulfide bridges, characterization for Fab- and Fc- region and carbohydrate content requirements remain unspecified.

*Immunological properties:* The antigen binding assay and epitope requirements are aligned with the EMA guideline. However, cytotoxicity evaluation, cross-reactivity and complementary ability evaluation remains undefined.

*Biological activity:* Relevant functional assays are indicated to be performed but details i.e. cytotoxicity (ADCC), cytotoxic properties (e.g., apoptosis), complement binding and activation ability, other effector functions i.e., Fc- gamma receptor binding activity and neonatal Fc- receptor (FcRn) binding activity remain undeclared.

*Purity, Impurity and Contaminants:* The fact sheets of BGTD/Canada demand purity testing for both the drug substance and the drug product. It also specifies identification, characterization and biological activity evaluation of impurities, if non-relevant with reference product. BGTD expects to have highly similar or same level (% of impurities) to comply with biosimilarity principle.

The general requirement for molecular heterogeneity is stated; however qualitative or quantitative nature of methods is not specified.

The requirements pertaining to multimers, aggregates and particulates are in line with those of the WHO, whereas contaminants are as per EMA requirements (Health-Canada/BGTD, 2017).

*Cell lines/ Quantity:* The expectation of cell lines as well as depth of information required in marketing application for biosimilar is yet to be spelled out. The views of BGTD/Canada for quantity determination of finished product need to be specified in the guideline.

*Specifications:* It is indicated that appropriate specification is to be chosen for the biosimilar, but further information such as determination of specifications, acceptance criteria, method of analysis and its validation and criteria for stability studies are not provided in detail (Health-Canada/BGTD, 2016).

##### BRICS-TM

Supplementary Table 7 reveals the comparative quality attributes for biosimilar development across BRICS-TM markets.

*ANVISA:* Resolution RDC n° 55/2010 specifies requirements pertaining to primary, secondary, tertiary and quaternary structure characterization; however immunological properties are not defined. The biological activity is expected to be determined, however requirement for type of assays are not defined. Impurity profile, process related impurities and contaminants are expected to be characterized. The other requirements for purity and heterogeneity are unclear. The criteria pertaining to cell lines, quantity and specifications are not defined (Anvisa, 2010).

*Russian Federation:* Russian Federation Law number 61-FZ on the circulation of medicines in Russia announces little about biosimilars. Hence characterization specifics for mAbs or any other biotechnological/ biological products are difficult to understand for manufacturers (Russian-federation, 2014).

*CDSCO:* The CDSCO India has established guidelines on similar biologics based on WHO guidelines. The requirements for mAb characterization remain in line with those of the WHO, whereas immunological property criteria remain unspecified. The biological assays are expected to be determined; however, the types of assays are not defined. Evaluation of multimers, aggregates and process related impurities are expected to be carried out. As to orthogonal methods for purity, contaminants, heterogeneity, these remain unspecified. The cell lines and quantity essentials are not spelled out. The qualified assays are required for characterization, however detailed requirements are unavailable (CDSCO, 2016).

*CFDA:* It is recommended to identify and characterize primary and advanced structure (secondary/tertiary/quaternary), structural heterogeneity and glycosylation. The expectations for immunological properties are to have comparative qualitative and quantitative analysis for Fab- and Fc- fragment including affinity for antigens, CDC and ADCC activity, affinity for FcRn, Fc gamma and C1q receptors. Purity to be determined in terms of hydrophobicity, charge and molecular size variant and various type of post translation modifications including glycosylation. Process impurities must be considered and new impurities to be characterized. For the biological activity it is expected that bioactivity test will be performed, consistent with the reference drug, however, details are not provided. The cell lines and quantity criteria remain undefined. The specifications should be consistent with the reference drug. It is expected that sensitive and advanced analytical techniques and methods will be used to detect potential differences between candidate drug and reference drug. It is recommended to use sensitive conditions for accelerated and forced degradation stability studies (CFDA, 2014).

*SAHPRA (previously known as MCC):* The biosimilar and reference product should be structurally, physico-chemically and biologically similar as per MCC guideline. The Medicines Control Council (MCC) issued biosimilar characterization requirement which remains unchanged with WHO/EMA. The immunological properties, cell lines and quantity criteria are undefined, whereas biological activities are expected to be characterized by both *in vitro* and *in vivo* assay(s). Heterogeneity and contaminants are not specified but are expected to have test performed for aggregate formation test and for quantifications of impurities. The process related antibody impurities should be considered. Validated analytical techniques are expected to be used for characterization (MCC, 2014).

*TMMDA:* Turkish draft biosimilar guideline is in parallel with EMA's overarching biosimilar guidelines. It is stated that this guideline could apply for any biological medicines. However, TMMDA has not published a specific guideline on mAbs yet.

According to the guideline, a physico-chemical characterization programme should include primary and higher order structures of the biosimilar. Any detected differences between the biosimilar and the reference medicinal product should be justified with respect to the micro-heterogeneous pattern of the reference product. The immunological functions of mAbs and related substances (e.g., fusion proteins based on IgG Fc) should be fully compared. This would normally include a comparison of affinity of the products to the intended target. In addition, binding affinity of the Fc to relevant receptors (FcγR, C1q, and FcRn) should be compared unless justified. Appropriate methodology should also be employed to compare the ability to induce Fab- and Fc-associated effector functions.

Biological assays using different and complementary approaches to measure the biological activity should be considered, as appropriate. Depending on the biological properties of the product, different assay formats can be used (e.g., ligand or receptor binding assays, enzymatic assays, cell-based assays, functional assays), taking into account their limitations. Complementary or orthogonal approaches should be followed to accommodate limitations regarding validation characteristics of single bioassays. State-of-the-art analytical technologies following existing guidelines and pharmacopoeial requirements should be applied to identify both of product-related and process-related substance and impurities and the potential risks related to these identified impurities (e.g. immunogenicity) will have to be appropriately documented and justified. The cell lines criteria remain unspecified. The rationale used to establish the proposed range of acceptance criteria for routine testing should be described. The claimed shelf life of the product should be justified with full stability data obtained with the biosimilar medicinal product. Comparative real-time, real-condition stability studies between the biosimilar and reference medicinal product are not required (General-Directorate-for-Pharmaceuticals-and-Pharmacy, 2017).

*COFEPRIS:* Though Mexico has used WHO Similar Biologic Products (SBP) guidelines as reference to establish Official Mexican Standard NOM-257-SSA1-2014, the guideline pertaining to mAbs biosimilar is not referenced. Hence no specific criteria for characterization are defined. The immunological properties, biological activity, purity, impurity, contaminants, cell lines, quantity and specifications are unclear, since no reference has been given to mAb specific EMA guideline (Cofepris, 2014).

##### Manufacturing process

Comparative characterization of manufacturing process would be challenging since reference product manufacturing process detail would be confidential. However, the agency's expectation for the manufacturing process is to produce targeted product having comparable molecular and quality profile. Apart from that, the process must be capable of manufacturing product of consistent quality. Appropriate in-process control parameters need to be defined at the time of process development. The platform manufacturing approach can be utilized with proper justification and evidence.

The process needs to be fully validated including validation of viral reduction study as per ICH Q5A; also, if material of animal origin is used then TSE guidelines are to be considered.

WHO recommends to optimize the process so as to minimize differences between SBP and RBP for reduced clinical testing and lesser impact on safety and efficacy (WHO-TRS, 2017).

BGTD expects that the applicant submits the proposed comparison in the manufacturing process to the reference biologic drug, in case such information is available (Health-Canada/BGTD, 2016).

##### Overages

Appropriate overages to be included based on variability of bioassay in *in vivo* condition.

##### Compatibility

It is required to perform compatibility between biological substance and excipients. The investigation of interaction study is mandatory if primary packing materials is different than reference product.

#### Non-clinical studies

WHO, EMA and FDA have described non-clinical evaluation in a stepwise manner and to be comparative. The primary objective of such studies is to explain differences observed in characterization studies.

As per EMA, WHO, BGTD/HC, CDSCO and TMMDA, *in vivo* non-clinical studies may be necessary, if characterization and *in vitro* studies warrant such studies (EMA, 2014a; CDSCO, 2016; Health-Canada/BGTD, 2016; General-Directorate-for-Pharmaceuticals-and-Pharmacy, 2017; WHO-TRS, 2017). According to USFDA limited animal toxicity data might be sufficient when comparative structural and functional data to prove analytical similarity (CBER, 2015).

ANVISA insists that comparative *in-vivo* non-clinical studies, covering pharmacodynamic studies and repeat-dose toxicity studies with toxicokinetic should be submitted, conducted in relevant species (Anvisa, 2010).

The determination of *in vivo* studies remains non-clarified with MCC/SAHPRA guideline, while COFEPRIS remains silent on non-clinical requirement (Cofepris, 2014).

##### In vitro studies EMA

Non-clinicfal studies should be performed in a step-wise manner, starting with comparative *in vitro* studies for binding and functional assays following with second step of additional *in vivo* studies, if necessary. The non-clinical studies should be performed on sufficient batches representing the proposed clinical trial batch. *In vitro* assays should be inclusive of binding target antigen(s) assay, binding assays with Fc gamma (FcγRI, FcγRII, FcγRIII) receptors, FcRn and complement (C1q), Fab- associated functions e.g. soluble ligand neutralization, activation or blockade of receptor, Fc-associated functions e.g., ADCC, CDC, complement activation, depending on the type of mAb.

It is expected to have comparative studies with capability to identify differences between the similar biological medicinal product and the reference medicinal product. In general, tissue cross-reactivity studies are not recommended. ADCC and CDC evaluation does not have to be performed for mAbs directed against non-membrane bound targets.

To decide *in vitro* studies should reference product information about receptors or antigens involved in pharmaco-toxicological and pharmaco-kinetics properties to be studied. Accordingly, comparative binding assays signal transduction and function activity/viability should be performed, and differences should be justified.

Based on the batch-to-batch variability and assay, the number of lots for reference product to be decided and justified. *In-vitro* studies should cover all attributes of mAbs, even though each of these not to be considered as essential for the therapeutic mode of action (EMA, 2012, 2014a).

##### WHO

In addition to EMA requirements, WHO has specified binding studies for antigen, Fc receptors and Fab- (i.e., neutralization of soluble ligand, receptor activation/blockade, reverse signaling via activation of membrane-bound antigen) and Fc- (i.e., ADCC, ADCP, CDC) associated functions (WHO-TRS, 2017).

##### USFDA

The pharmacologic activity of protein products should be evaluated by *in vitro* functional assays such as biological assays, binding assays, and enzyme kinetics. These assays should be comparative thus they can provide evidence of similarity or reveal differences in the performance of the biosimilar. The requirements are similar those of the EMA and those of the WHO (CBER, 1997).

##### Health Canada/BGTD

Non-clinical *in* vitro studies are recommended, however no further details i.e. of binding target antigen(s) assay, binding assays with Fc gamma receptors, FcRn and complement (C1q), Fab- and Fc-associated functions, are available (Health-Canada/BGTD, 2016).

##### BRICS-TM

The *in vitro* assay related information is not defined in the guideline published by ANVISA, the Russian federation, CFDA and COFEPRIS (Anvisa, 2010; CFDA, 2014; Cofepris, 2014; Russian-federation, 2014). The CDSCO defines i*n vitro* cell based bioassays i.e. cell proliferation/ cytotoxicity/ neutralizing/ receptor binding assays to be performed as part of *in vitro* studies (CDSCO, 2016). The requirements for SAHPRA and TMMDA are defined as in line with EMA's overarching biosimilar guidelines (General-Directorate-for-Pharmaceuticals-and-Pharmacy, 2017).

##### In vivo studies EMA

For biological products, *in vivo* studies are to be performed if *in vitro* studies show significant differences (i.e. new/modified structure, quantitative difference in quality attributes, formulation difference etc.) between the proposed biological product and the reference product. Differences in structure/quantity/formulation (novel excipient) /inherent nature of molecule, presence of relevant quality attributes different from the reference product might become a key deciding parameter for *in vivo* studies. For additional information transgenic animal/transplant models can be considered. Direct human studies can be done if a relevant animal model is unavailable. The duration of the study should be justified based on PK behavior of the mAb and its clinical use.

*PKPD:* The dose concentration-response assessment studies need to be performed considering targeted human dose and quantitatively compared with reference product. The study needs to be performed as per Article 4 of Directive 2010/63/EU.

*Repeated-dose toxicity:* It is expected that a flexible approach is considered only if non-human primate is the relevant species. Comparative studies with inclusion of one single dose of reference product and biosimilar and/or one gender and/or no recovery animals/evaluation of in-life safety parameters need to be performed. The highest dose of the range can be selected for one-dose evaluation and justification should be given accordingly. EMA does not recommend repeat dose toxicity study in non-relevant species to assess non-specific toxicity to justify differences in production processes.

*Immunogenicity:* Though animal immunogenicity is not predictive in human, blood samples should be taken for future pharmaco/toxico kinetic evaluation.

*Safety Pharmacology, Reproduction Toxicology and Carcinogenicity*: Not required.

*Local tolerance:* Required only if novel excipients for proposed ROA are used and such study can be part of other *in vivo* studies (EMA, 2007, 2014a).

##### WHO

*PK and/or PD:* Comparative study including intended exposure in humans must be performed via dose-response assessment, if a relevant animal model is available.

*Repeat-dose toxicity study:* It is not recommended in non-human primates. The highest known dose needs to be selected if quantitative differences are required for evaluation. For qualitative differences, the relevant doses should be selected so that potential differences between RBP and SBP can be identified. Antibody measurements should be included to support toxico-kinetic data interpretation and the overall comparability exercise.

*Immunogenicity study:* WHO has advised to withdraw blood samples in case this study is required for interpreting PK/TK.

*Local tolerance studies:* to be evaluated depending upon route of administration and can be carried out as part of repeat-dose toxicity study.

Safety pharmacology, reproductive toxicology, genotoxicity and carcinogenicity studies are generally not required unless justified based on RBP properties (WHO-TRS, 2013).

##### USFDA

*In vivo* studies are advised to be performed in line with preclinical safety evaluation of biotechnology-derived pharmaceuticals S6 (R1) (CBER, 1997; EMA, 2007). The pharmacologic activity of protein products should be evaluated by *in vitro* functional assays as well. Animal toxicity studies are considered useful if there are uncertainties about the safety of a biosimilar product after extensive structural and functional characterization. The scope and extent of animal toxicity data is dependent upon information on the reference product, biosimilar product and known similarities and differences between the two.

It is strongly recommended to discuss with the Agency for not conducting animal studies or scope and extent of these studies (CBER, 2018). Even though animal PK and PD studies were conducted, the need for human PK and PD studies remains. Animal immunogenicity assessment helps to interpret animal study results but generally do not predict potential immune response in human.

##### Health Canada (HC)/BGTD

The guideline indicates that *in vivo* studies are not required, if similarity is proven in the previous steps. Details about *in vivo* criteria, if required, are unavailable (Health-Canada/BGTD, 2016).

##### BRICS-TM

**Figure 3:**
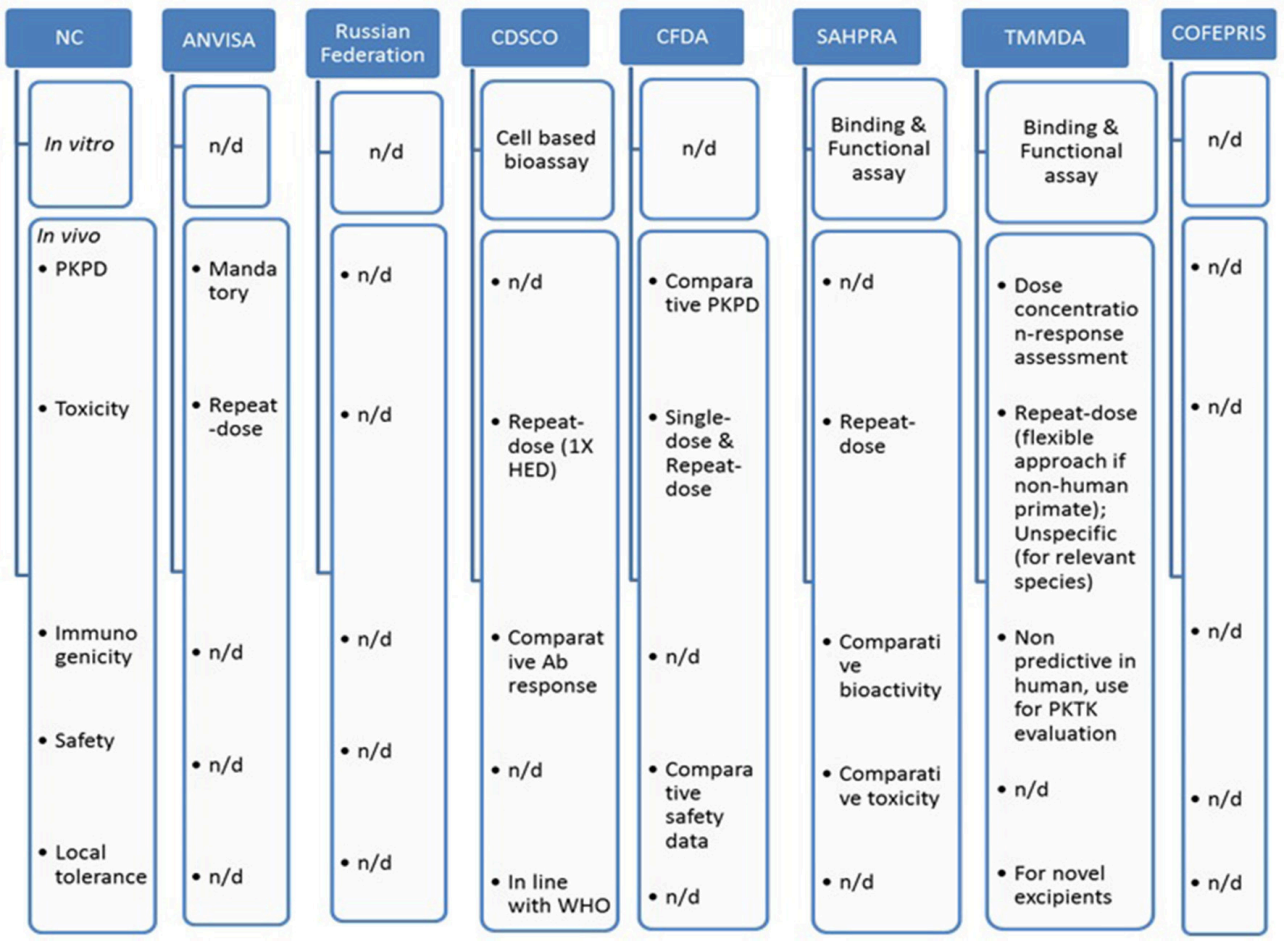
Comparative non-clinical attributes across BRICS-TM markets. n/d, not defined; HED, human equivalent dose; PKTK, pharmacokinetics toxicokinetics.

Figure 3 indicates comparative nonclinical (*in vitro* and *in vivo*) requirements across BRICS-TM markets.

PD studies for intended therapeutic indication and repeat-dose toxicity studies including toxicokinetic in relevant species are mandatory for ANVISA; however detailed requirements are not specified (Anvisa, 2010). Such study information is unavailable from the Russian federation and COFEPRIS (Cofepris, 2014; Russian-federation, 2014).

The SAHPRA guideline states that *in vivo* animal studies, with at least one repeat-dose toxicity study with toxico-kinetic measurements is required to be presented to show comparative toxicity and bioactivity. Such study should analyze immunogenicity as well as relevant specific safety concerns. Other toxicological studies are not required unless needed, based on reference product (MCC, 2014).

*In vivo* studies may not be required if similarity is proven in the previous steps as stated in CDSCO guideline. A repeat-dose toxicity study is recommended to be performed, at minimum one repeat-dose study, with at least 1X of human equivalent dose (HED), in a relevant animal model, with intended ROA, for not < 28 days with 14 days recovery period. For a pharmacologically relevant animal model, the intended route of administration should be included. In case a relevant model is not available, studies should be performed in two species i.e., one rodent and other non-rodent and Schedule Y should be followed for route of administration. Moving further to immunogenicity, the CDSCO requires comparative antibody responses in a suitable animal model study. It should be part of sub-chronic repeated-dose study. The local tolerance studies and other toxicological studies requirements are aligned with WHO, whereas safety pharmacology, reproductive toxicology, mutagenicity and carcinogenicity studies are generally not required unless justified based on the RBP's properties (CDSCO, 2016).

The CFDA needs comparative non-clinical *in vivo* studies as far as possible. Single dose and repeat-dose toxicity studies are essential to be performed (CFDA, 2014).

The TMMDA requirements are defined as in line with EMA's overarching biosimilar guidelines (General-Directorate-for-Pharmaceuticals-and-Pharmacy, 2017).

#### Clinical studies

##### Pharmacokinetics and Pharmacodynamics studies EMA

*Pharmacokinetics (PK):* The clinical data need to be obtained using the proposed biosimilar product, from a commercial batch, to ensure similarity of quality profile between comparability and commercialized batch.

Comparative PK is expected, and comparability needs to be proved considering clearance and/or half-life of therapeutic protein. Methods used for analyzing immunoassays and bioassays should be validated. The bio-analytical method should be capable of identifying and analysing the parent molecule and/or degradants, if any.

PK studies should be performed in healthy volunteers, screened for homogeneity to perform single-dose study. The preference is single-dose, cross-over with PK profile characterization including late elimination phase. Parallel group design can be explored for longer half-life and/or higher immunogenicity risk. When PK studies are performed in healthy volunteers, data needs to be extrapolated to target population. In case healthy volunteers are not possible to enroll in single-dose PK studies, then patients can be used in multi-dose PK study. The relative bioavailability needs to be investigated for individual administration site. The dose proportionality needs to be evaluated in single or multiple doses with discussion of clinical impact. Studies to be performed at several dose levels and occasions. PK/PD relationship needs to be established and evaluated.

*Disease and patient characteristics:* The age of patient population, manifestation of disease at specified age, the type of different treatments taken by the patients play important role in selecting patients. It is advisable to perform comparative PK study in mono-therapy to reduce variability. It is recommended to select patient in better clinical condition, wherein the mAb can be given in combination with other therapies.

*PK characteristics of reference mAb:* The designing of anticancer mAb should be evaluated by considering multiple dosing. Based on elimination mechanism (target mediated/non-target mediated) of mAb, the PK studies should be designed. If it is eliminated by both ways, comparable PK in healthy volunteers should be performed for non-target mediated whereas the other one should be performed in patient population as support data. If receptor shedding is involved, then baseline comparability studies should be performed. PK profile is not required for all conditions specified for license mAb unless therapeutic category is different.

*Doses:* The lowest therapeutic dose in patients should be sufficient enough to identify difference in target mediated clearance.

*Route of administration:* Subcutaneous routes should be sufficient since this will characterize absorption and elimination.

*Sampling:* To be selected at first and last administration for single dose study whereas for multiple dose study steady state sampling is most preferred.

Design- A single-dose, cross-over with PK profile characterization including late elimination phase is most preferred. Parallel group design can be explored for longer half-life and/or higher immunogenicity risk.

Regarding the route of administration, if two different routes such as intravenous and subcutaneous are assigned to the reference product, comparability PK study with subcutaneous route alone would be sufficient with justification.

Acceptable range should be based on clinical judgment.

Parameters to be estimated as below:

Primary parameter should be AUC _(0−inf)_ in single dose study. C_max_, T_max_, volume of distribution and half-life and other secondary parameters should be estimated and for subcutaneous administration C_max_ should be co-primary parameter.

As to multiple-dose study primary truncated AUC after first until second administration AUC_(0−t)_ and steady state AUC over dosage interval, secondary- At steady state C_max_ and C_trough_ should be primary parameters.

*Pharmacodynamics (PD):* For mAb PD studies, two types of possibilities exist depending upon the availability of PD markers. One in which PK and PD can be combined together if PD marker is available. In case PD markers are not available *in vitro* testing should be performed.

To prove comparability dose concentration response relationship or time response relationship should be established.

If fingerprinting approach is used by selecting non-surrogate PD markers, then advice from regulatory agency is essential.

*Clinical efficacy:* The clinical efficacy studies are carried out to establish that the biosimilar will not perform in a manner that differs from the originator in clinically significant manner. The principle is to demonstrate similar efficacy compared to the reference medicinal product, not only patient benefit which is already proven by the reference medicinal product.

*Study type:* parallel design, random, double-blind adequately powered comparative clinical studies using efficacy end-points in absence of surrogate markers for efficacy.

*Population:* Patient for approved therapeutic indication of reference product, in case of unapproved indication, justification should be provided.

*Design:* Equivalence design expected, non-inferiority design needs agency's consent.

*Endpoints:* secondary endpoints of reference product are sufficient for comparison with reference product.

*Comparability margin:* To be justified with some statistical and clinical grounds by considering assay sensitivity.

The clinical studies in pediatric and elderly population are not required. The inclusion of patients from non-European countries may increase heterogeneity but if there are no known intrinsic differences then it is possible to include mixed populations.

*Clinical safety;* Comparative safety data is expected to be obtained before product authorization and the follow-up period chosen needs to be justified. The adverse reactions are required to be compared in terms of type, frequency and severity.

Immunogenicity and other risks need to be evaluated and incorporated into the application dossier. Increased immunogenicity as compared to reference product may lead to products not considered to be truly biosimilar. Double-blind, parallel analysis need to be done for measuring immunogenicity. The analytical assays should have capability to detect reference and proposed product antibodies and in addition all possible antibody of proposed product. The antibody titers, cross-reactivity, targeted epitopes and antigen neutralizing capacity are required to be determined. The immunogenicity study duration could be minimum 4 weeks in case of immune suppressive agents are used or otherwise justified based on treatment duration and removal of product from the circulation as well as start of humoral immune response. Follow-up duration of 6 months during pre-authorization studies can be justified based on immunogenicity profile and for chronic 1-year follow-up data before marketing authorization application is essential. Further follow-up data can be submitted post-authorization.

*Extrapolation to other indications:* Extrapolation (or extension of the indication) is considered based on scientific justification of quality (physico-chemical, structural and *in vitro* function test), non-clinical (PK/PD) and clinical (safety/efficacy) comparability in one indication.

It might be challenging to extrapolate if the active substance acts on several or multiple receptors with different clinical outcomes in varied indications or has more than one active site or the studied indication is irrelevant in terms of safety and efficacy to the other indications.

Extrapolation of immunogenicity to other indications would require justification (based on whether the therapy is one agent, or the biological is added to another immunosuppressant.

*Pharmacovigilance (PV) and Risk management plan (RMP):* Pharmacovigilance system details will be required and fully described in marketing authorization applications. Suspected adverse reactions will need appropriate tracing with brand name and batch details of each product.

A Risk management plan, defining all known and potential unknown risks needs to be monitored post market authorization of the product. In addition, the safety studies of biosimilars should cover all ongoing safety expectation of the reference product (EMA, 2014a).

##### WHO

*PK*: Apart from EMA's recommendation for using commercial batch proposed product for clinical studies, WHO insists on PK bridging studies providing PK profile comparison between two different formulations, if non-commercialized product is used.

Single-dose pharmacokinetic data studies are sufficient in general, however for mAbs, parallel group design with a larger number of subjects are required (due to long half-life of mAbs, single-dose, cross-over design may be inappropriate).

Key issues to be considered for mAb PK:

1. Healthy subject to be used due to higher sensitivity and homogeneity in comparison to patient population
2. Sub-therapeutic dose to be considered due to ethical issues
3. Study in patient population could be mandatory due to safety risks in healthy volunteers
4. It may be necessary to perform a PK study in a different population considering different therapeutic indications under development.

Antigen/receptor level, presence of target-mediated clearance and/or receptor shedding of reference mAb has to be considered for selection of the population to be studied.

Drug interactions and special population studies are not required. For mAb biosimilar, it is not required to perform PK study for each authorized indication of reference biological product.

It is expected that comparative PK for mAbs will be performed, considering mono-therapy to reduce variability source. However comparative PK for both mono and combination therapies have to be considered, if concomitant therapy alters the PK of the mAb considered as a biosimilar.

Lowest recommended therapeutic dose needs to be used for PK profiling. A higher dose may be required based on mAb clearance characteristics.

To measure C_max_, sufficient sampling is expected at early time points. For single-dose study, sampling has to be done till last quantifiable concentration available. In multi-dose studies sampling has to be done at first dose and at steady state (expected to reach after 5 half-lives of mAb). However, there is no clarity about type of 5 half-lives as PK half-life or biological half-life.

Equivalence margin- For primary parameters 80-125% of comparability margin is acceptable with justification.

*PD:* WHO recommends including PD markers as part of clinical comparability exercise or confirmatory PD studies may be performed in place of clinical safety or efficacy studies.

*Clinical efficacy:* Design**:** An equivalence trial design is expected, with emphasis on the additional benefits for extrapolation of indications.

The rest of the requirements for efficacy trials are equivalent to those of the EMA.

*Clinical safety:* The comparative clinical safety requirement is in line with EMA's guideline; however, WHO presents a multi-disciplinary approach for evaluation of immunogenicity in mAbs. It covers risk assessment, risk-based immunogenicity programme, comparative immunogenicity, assays and mAb characterization and clinical immunogenicity assessment.

*Extrapolation to other indications:* The extrapolation to other indications can be considered subject to usage of a sensitive clinical model for identification of differences, the mechanism of action and/or applicable receptors are the same, no new safety issues are expected when extrapolating indications and equivalence design efficacy trials have been performed.

*Pharmacovigilance (PV) and Risk management plan (RMP):* WHO has advised to refer to ICH E2E for PV planning. In general PV requirements are according to EMA's expectations (WHO-TRS, 2013).

##### USFDA

*clinical pharmacology:* The PK and PD response assessment, evaluation of residual uncertainty and analytical quality and similarities are defined as three basic concepts for proposed biosimilar development. To access potentially meaningful difference between reference product and proposed biosimilar, the inclusion of exposure and wherever possible exposure response is essential in designing clinical PK/PD study. To evaluate clinical pharmacology similarity, inclusion of PK similarity and PD similarity (if applicable) are essential.

The PD response can be measured by using a single or a composite biomarker.

It is expected to use material from the final manufacturing process when performing a clinical pharmacology study. Analytical and PK bridging study with the to-be-marketed product will be necessary in case material is used from different manufacturing processes.

The PK study design is recommended as cross-over for short half-life product, having rapid PD onset and lower expected immunogenicity. The PD assessment has to be performed using multi-dose as against single dose of the PK study. The products with long half-life, requiring repeated doses and chances of increased immune responses will require parallel design studies.

Healthy subjects are expected if product can be administered safely. However, patient population can be chosen if there are ethical challenges or there are available PD markers in patients (CBER, 2016).

If drug-drug interaction and QT/QTc prolongation and proarrhythmic potential studies are on-going for BLA holder, then such studies would be essential for biosimilar manufacturer as part of post-marketing approval (CBER, 2015).

It is expected to select the most sensitive dose for evaluation of PK/PD similarity; based on the condition of the patient, the dosing regimen can be decided.

The route of administration of the proposed product should be the same as that of the reference product.

PK measurement: peak of concentration (C_max_), the total area under the curve (AUC) for the reference product and proposed biosimilar. For single dose study AUC to be calculated as AUC _(0−∞)._ For multiple dose study the total exposure to be calculated as time concentration profile starting from zero to end of dosing interval, at steady state, as AUC_(0−tau)_.

The average equivalence statistical approach is expected to compare clinical PK and PD similarity. To prove similarity the expected calculated confidence interval limit is 80-125% if the limit varies then justification is expected.

With reference to safety and immunogenicity, the data is expected to be collected from clinical PK and PD study.

To evaluate clinical pharmacology similarity, FDA recommends three types of bio-analytical assays as ligand binding assays, concentration and binding assay, and PD assay (CBER, 2016).

*Extrapolation of indications:* The proposed biosimilar product can be licensed for additional indications, provided one indication which is approved for the reference product was the subject of biosimilarity studies and biosimilarity has been proven. In addition, there needs to be consideration on whether the clinical study performed scientifically justifies the extrapolation. Apart from that, extrapolation of indications in pediatric population is also possible, subject to scientific justification.

The issues pertaining to MOA, PK and bio-distribution in varied patient populations, immunogenicity, anticipated toxicity differences and other relevant factors impacting efficacy should be scientifically justified for the tested and all other extrapolated indications (CBER, 2015).

*Risk evaluation and mitigation strategy (REMS):* REMS in line with reference product are required to be submitted for proposed biosimilar product (CBER, 2015).

##### Health Canada (HC)/BGTD

*PK:* The guidance document pertaining to information and submission requirements for biosimilar biologic drugs indicates PK requirements. It covers comparative PK study at low or sub-therapeutic dosage in healthy subject or patients with appropriate justifications. The design of the study needs to be decided, based on set parameters. The equivalence margin for primary parameters is expected as 90–125%.

The guidance document also states that comparative PK criteria are to be defined based on the bioequivalence guidance document: Conduct and analysis of comparative bioavailability studies and comparative bioavailability standards: Formulations used for systemic effects. However, the said guidance document excludes applicability to subsequent entry biologics under scope.

*PD:* The guidance recommends characterizing the PK/PD relationship if both studies are combined. Apart from equivalence trial expectation, PD markers can be used, subject to justification. For Canada, the calculations for PK studies should follow those outlined for Bioequivalence studies as in the Guidelines for Generic products. Acceptability of fingerprinting approach is unclear with BGTD/Canada (Health-Canada/BGTD, 2016).

*Clinical efficacy:* Comparative clinical trials with equivalence design are expected.

*Clinical safety:* Comparative clinical safety data (in terms of adverse events including nature, severity and frequency) in sufficient number of patients treated for suitable duration is required as part of biosimilar application.

The BGTD has not defined immunogenicity requirements in precise manner but accepts what is submitted provided it is clearly laid out, well explained and justified. In general, the expectations for comparative immunogenicity studies are aligned with those of the WHO (Health-Canada/BGTD, 2012). The follow-up duration for pre and post-authorization study is not defined precisely however agency accepts what is submitted if the plan is clearly laid out, well explained and justified.

*Extrapolation to other indications:* All indications can be authorized based on one indication, subject to provision of scientific rationale. However, if reference product's specific indication is not approved in Canada, then extrapolation may not be possible.

*Pharmacovigilance (PV) and Risk management plan (RMP):* A Risk management plan needs to be prepared in consultation with BGTD. In general, it covers requirements mentioned in the EMA guideline and/or specific guidance document- Submission of risk management plans and follow-up commitments published by Health Canada/BGTD (Health-Canada/BGTD, 2016).

##### BRICS-TM

Supplementary Table 8 indicates clinical requirements across BRICS-TM markets.

ANVISA states comparative PK study along with PD can be performed: however, there is no detailed information available in Resolution RDC n° 55/2010. Comparative clinical studies are required to be performed for safety and efficacy: however, no further details have been provided. Extrapolation to other indications is possible if product is registered through route of development (and not through individual route of development), however further detail is not defined. RDC n° 55/2010 does not specifies detailed requirements on PV and RMP: however, it refers to health legislation in effect (Anvisa, 2010). The Resolution does not address the question of interchangeability (Castaneda-Hernandez et al., 2014).

The biologic/mAb specific information is unavailable in the Russian guideline (Russian-federation, 2014).

With respect to CDSCO, the PK study requirements are aligned with those of the WHO. Comparative, parallel/cross-over, healthy volunteers/patients, PD study is recommended if at least one PD marker is linked with efficacy, which is well characterized for the reference biologic. If PK study can be done in patients and PD marker is not available, then PK and PD studies can be combined in a phase III clinical trial. Confirmatory safety and efficacy are mandatory for similar biologics. Equivalence, non-inferiority or comparability phase III clinical trials can be conducted. Trial population size can be reduced if a similar biologic is indicated for rare diseases. Comparative safety study to be performed based on adverse events, nature, severity and frequency. It is stated that immunogenicity data should be obtained in PK/PD studies, if a phase III trial is waived. No further details were available for immunogenicity studies. Pre and post approval safety assessment data are required. If safety and immunogenicity studies are performed in more than 100 patients during pre-approval, phase IV studies, patient numbers can be reduced accordingly. Based on one specific clinical indication comparability data, extrapolation can be done to other indications subject to the same mechanism of action and receptors for all indications. New indications of innovator can be approved by separate application. As per PV plan, periodic safety update reports (PSURs) to be submitted every 6 months for initial periods post approval of similar biologics. Annual PSURs to be submitted for the subsequent 2 years (CDSCO, 2016).

The CFDA guideline about PK studies states healthy volunteers or patients, design of the study, single/multiple dose study and equivalence design with inclusion of elimination characteristics, however details pertaining to similarity criteria, dose selection sampling parameters are unavailable. Comparative PD studies and PD biomarker usage are indicated with no further detailed information. Comparative efficacy trial study has to be performed, if clinical study material of proposed product is different than the commercially available product. PKPD requirements are aligned with the WHO. It states that only common adverse reaction is to be compared and tested, whereas information pertaining to unknown safety parameters is not defined. Comparative clinical immunogenicity studies can be conducted as part of PK, PD and/or efficacy trials and considered for detecting antibodies linked to process-related impurities. Extrapolation of indications can be considered based on scientific justification. PV and RMP (safety and immunogenicity) have to be submitted and evaluated as per national regulations (CFDA, 2014).

SAHPRA recommends PK study requirements similar to EMA guideline on the clinical investigation of pharmacokinetics of therapeutic proteins. It recommends combined PK/PD studies, PD marker determination, selection of design and duration, all based on justification. Comparative PD studies in a justified population are acceptable. Comparative PK/PD studies may be sufficient for clinical comparability if predefined conditions are met. Comparable clinical efficacy trials should be conducted. If a clinical comparability trial design is not feasible, other designs should be explored. Safety and immunogenicity need to be sufficiently characterized. Pre-registration of safety data has to be performed in sufficient number of patients. The basic principle for performing immunogenicity studies is in line with EMA and WHO guidelines. If non-inferiority comparability studies have been conducted for one indication, extrapolation to other indications is possible. PV has to be based on MCC guidelines and RMP should be presented or planned at the time of marketing authorization application (MCC, 2014).

The TMMDA requirements are defined as in line with EMA's overarching biosimilar guideline for clinical studies (General-Directorate-for-Pharmaceuticals-and-Pharmacy, 2017).

PK/PD, Clinical criteria are not defined; however, PV information needs to be submitted in line with Mexican standard (Cofepris, 2014). As indicated in regulations for biosimilars in Latin America, Europe and India, review article by Castaneda Hernandez, the Mexican regulations do not permit extrapolation between indications (Castaneda-Hernandez et al., 2014).

## Discussion

The mAbs are complex biotherapeutic products requiring stepwise and comprehensive development strategy considering quality, non-clinical and clinical aspects to obtain biosimilar approval by mature and emerging regulatory agencies. The biosimilarity principles including development approach, basis of biosimilarity, demonstration of biosimilarity with reference product and type of applications is quite uniform across EMA, WHO and USFDA. However, with respect to BRICS-TM, some of the parameters i.e. the simplified approach is yet to be clarified by Brazil (ANVISA), China (CFDA), Russian federation and Mexico (COFEPRIS).

The parameters such as improved efficacy/safety, comparability studies for post-approval changes and interchangeability await clarifications from BRIC agencies. The SAHPRA disallow interchangeability/switching whereas TMMDA leaves the decisions with medical practitioners. The guidance for pediatric research is at a very primitive stage across BRICS-TM.

In general, mature agencies expect the reference product to be sourced from their own territory having licensed the product, based on full development data and provision of bridging data for selection of non-reference product, for certain comparability studies. It is evident that non-authorized reference product selection criteria are missing for Russia and China whereas Turkey does not require non-reference product for characterization. The rest of BRICS-TM agencies allow selection from ICH/own aligning countries while Mexico allows biosimilar product to be used as reference product. Surprisingly, none of the emerging market agencies talks about bridging data.

The comparative characterization exercise of the proposed biosimilar in relation to the reference product specifies physico-chemical studies (including detailed mAb structure, immunological properties, biological activity, purity impurity and contaminants, cell lines, quantity, specification), manufacturing process, overages and compatibility as detailed in the EMA guidelines. Although WHO has broadly covered all four components of characterization, some of the points i.e. amino acid sequencing, groups and bridges determination, cytotoxicity, CDR and epitope evaluation, multimers and aggregate characterization, contaminants control, immortalization approach and need for experimental/in-process stability data requirements are not specified. Most of the NRAs in emerging markets have prepared similar biologic guideline based on those of the WHO, hence presence/absence of data requirements are similar to the ones of the WHO. The requirements pertaining to manufacturing process, overages and compatibility characterization are not covered in the BRICS-TM guidelines except a reference to WHO or ICH Q5A. The mAb characterization requirement of USFDA is aligned with ICH member states. In the view of the authors, it would be useful for BGTD to revisit and define criteria for physico-chemical characterization.

The comparative *in vitro* assays for binding signal transduction and functional activity/viability studies are specifically defined by the WHO, whereas reference guidelines of BRICS-TM markets (except India), do not specify the need.

The repeat-dose toxicity, local tolerance, safety pharmacology, reproductive toxicity, carcinogenicity *in vivo* toxicity studies are aligned with EMA, WHO, TMMDA and SAHPRA. The USFDA has considered ICH S6 (R1) whereas BGTD lacks detailed information except non-requirement of *in vivo* studies, if similarity is proven in previous steps. Russia and Mexico do not specify detailed requirements whereas South Africa and India are equivalent to the WHO. The immunogenicity toxicity studies are essential for SAHPRA whereas EMA and WHO recommend withdrawal of samples for PK/TK interpretation. The detailed guidance for immunogenicity is awaited from other agencies.

The usage of commercial batch supply for clinical study is the same for EMA and WHO, whereas WHO defines the performance of bridging PK studies, if two different formulations are used. The PK study is expected to be a single dose, parallel design with late elimination stage by both agencies for mAbs. The WHO expects 80–125% of comparison margin for primary parameters. There are no specific PK criteria defined for ANVISA, Russian federation and COFEPRIS whereas CDSCO is aligned with WHO and TMMDA (Turkey) with EMA. Canada advises to refer the comparative BA studies and standards; however, BA standards exclude applicability to subsequent entry biologics under scope because BA guidance refers to oral and not injectable preparations. Although the standards for analytical results still apply to subsequent entry biologics. Comparative clinical safety or efficacy could be enough, subject to PD marker has been incorporated in PK/PD studies, with EMA, WHO, BGTD, CDSCO, TMMDA, CFDA and SAHPRA. As indicated earlier, Russia, and Mexico do not specify detailed PD requirements.

Efficacy trials are required as parallel design, random, double-blind, adequately powered with efficacy endpoint. Equivalence trials are expected, and non-inferiority needs the consent of the agencies. The efficacy trial design and type remain the same for EMA, WHO, BGTD, TMMDA and CFDA. No detailed information is available for ANVISA, Russian federation and COFEPRIS. Confirmatory Phase III clinical safety and efficacy are mandatory for India, whereas South Africa allows trial design to be explored in case a comparative clinical trial is not feasible. Comparative clinical safety data need to be obtained before authorization and follow-up data to be submitted post authorization across agencies stated in this article, except Brazil, Russia, and Mexico. The extrapolation of one indication to others is acceptable based on scientific justification except with Mexico. Interchangeability, switching and substitution of biosimilars is not defined in BRIC whereas South Africa restricts, as per section 22F (Generics substitution) Act 101 of 1965. TMMDA leaves interchangeability decision to practitioners. Pharmacovigilance and RMP data need to be submitted across agencies.

To conclude, it is evident that regulatory frameworks for the market authorization of biosimilars pose multiple challenges to the companies in the emerging markets (Gautam, 2017). The mAb specific development guidelines are yet to be published by BRICS-TM agencies. The current Russian federation law FZ-61 (December, 2014) has incorporated a definition for biological/biosimilars, however the roles of the expertise are to be determined by concerned federal authorities, resulting in unfamiliar regulatory framework (Lozda, 2016), the regulatory pathway would need clinical trial requirement based on complexity of molecule (Priori, 2013). Mexico (COFEPRIS) is yet to come up with detailed clarification for characterization, non-clinical and clinical comparability criteria. Though there are gaps in mAb biosimilar regulatory guidelines in emerging markets, we believe that the agencies are working hard to align regulatory norms in line with well-established agencies. The regulator participates in multiple forums, exchanges knowledge and is willing to upgrade. It would be advisable for the companies to approach agencies in advance to obtain biosimilar development advice so that hassle free authorization can be obtained. Further to this, the authors are working on verifying gaps in biosimilar development, regulatory evaluation procedures and approval matrices from BRICS-TM agencies and will present in their subsequent publications.

## Author contributions

SS developed the concept, provided guidance, coordinated the research and reviewed the first draft of the manuscript. HR retrieved, reviewed, analyzed, interpreted, and prepared the overview, similarities and differences between EU, WHO, Canada, Brazil, Russia, India, China and Mexico guidelines and wrote the first draft of the manuscript. HC retrieved, reviewed, analyzed, interpreted and prepared an overview regarding similarities and differences between Turkey, EU, WHO, USA and South African guidelines and wrote the first draft of the manuscript.

### Conflict of interest statement

The authors declare that the research was conducted in the absence of any commercial or financial relationships that could be construed as a potential conflict of interest.

## Abbreviations

ADCC: Antibody-Dependent Cell-Mediated Cytotoxicity
ADCP: Antibody-Dependent Cellular-Phagocytosis
ANVISA: National Health Surveillance Agency
AUC: Area Under Curve
AUEC: Area Under The Effect Curve
BGTD: Biologics and Genetic Therapies Directorate
BRICS-TM: Brazil Russia India China South Africa- Turkey Mexico
CDC: Complement Dependent Cytotoxicity
CDE, Center of Drug Evaluation, CDR: Complementarity-Determining Regions
CDSCO: Central Drug Standards Control Organization
CFDA: China Food and Drug Administration
CHMP: Committee for Medicinal Products for Human Use
COFEPRIS-: Federal Commission for the Protection Against Sanitary Risks
EEA: European Economic Area
EMA: European Medicines Agency
Fc: fragment crystallizable
FcRn: neonatal Fc receptor
HC: Health Canada
HED: human equivalent dose
mAbs: monoclonal antibodies
MOA: Mechanism of Action
NRA: National Regulatory Authority
PMS: Post Marketing Surveillance
PK: Pharmacokinetic
PREA: Pediatric Research Equity Act
PSUR: Periodic Safety Update Reports
PV: Pharmacovigilance
RBP: Reference Biologic Product
RMP: Risk Management Plan
ROA: Route of Administration
SAHPRA: South African Health Products Regulatory Agency
SBP: Similar Biologic Product
TK: Toxicokinetic
TSE, Transmissible Spongiform Encephalopathy, USFDA: United States Food & Drug Administration
WHO: World Health Organization.
