# Supplementary material for: Quality, Non-clinical and Clinical Considerations for Biosimilar Monoclonal Antibody Development: EU, WHO, USA, Canada, and BRICS-TM Regulatory Guidelines
Source: Front Pharmacol. 2018 Sep 28;9:1079. doi: 10.3389/fphar.2018.01079 (PMC6192287; doi:10.3389/fphar.2018.01079)
Supplement: Supplementary file 1 [file Data_Sheet_1.PDF]

Table 1: List of authorized mAb biosimilars in each country between 2013-2018

Year of MA	EMA	USFDA	HC/BGTD	ANVISA	Russian federation	CDSCO	SAHPRA	TMMDA	COFEPRIS
**2018**									
Adalimumab	*Halimatoz (Sandoz GmbH)	-	-	-	-	Mabura (Hetero)	-		-
	*Hefiya (Sandoz GmbH)	-	-	-	-	-	-	-	-
Bevacizumab	(Mvasi Amgen Europe B.V.)	-	-	-	-	-	-	-	-
Infliximab	Zessly (Sandoz GmbH)	-	-	-	Remicade (BIOCAD)	-	-	-	-
Trastuzumab	Trazimera (Pfizer Europe MA EEIG)	-	-	-	-	Hervycta (Dr Reddy's laboratories (DRL))	-	-	-
	Kanjinti (Amgen Europe B.V, Breda)	-	-	-	-	-	-	-	-
	Herzuma (Celltrion Healthcare Hungary Kft)	-	-	-	-	-	-	-	-

Rituximab	-	-	-	-	-	-	-	Redditux Trpharm Ilac San. Tic.A.S	-
**2017**									
Trastuzumab	Ontruzant (Samsung Bioepis UK Limited)	Ogivri (Mylan GmbH)	-	Zedora (Biocon-Mylan)	-	-	-	-	-
Adalimumab	Cyltezo (Boehringer Ingelheim International GmbH)	Cyltezo (Boehringer Ingelheim)	Hadlima & Hadlima Pushtouch (Samsung Bioepis Co Ltd)	-	-	-	-	-	-
	Imraldi (adalimumab) Samsung Bioepis UK Limited	-	-	-	-	-	-	-	-
	****Amgevita (adalimumab) Amgen Europe B. V.	-	-	-	-	-	-	-	-
	****Solymbic (adalimumab) Amgen Europe B.V.	-	--	-	-	-	-	-	-
Rituximab	Blitzima (Celltrion Healthcare Hungary Kft.)	-	-	-	-	Acellbia (BIOCAD)	-	-	-

	Ritemvia (rituximab) Celltrion Healthcare Hungary Kft.	-	-	-	-	-	-	-	-
	***Rituzena (previously Tuxella) (rituximab) Celltrion Healthcare Hungary Kft.	-	-	-	-	-	-	-	-
	***Truxima (rituximab) Celltrion Healthcare Hungary Kft.	-	-	-	-	-	-	-	-
	**Rixathon (rituximab) Sandoz GmbH	-	-	-	-	-	-	-	-
	**Riximyo (rituximab) Sandoz GmbH	-	-	-	-	-	-	-	-
Etanercept	Erelzi (Sandoz GmbH)	-	-	Brenzys Cristalia Brazil	-	-	-	-	-
Bevacizumab	-	Mvasi Amgen-Allergen	-	-	-	Krabeva Biocon	-	-	-
Infliximab	-	Renflexis (Merck Sharp and Dohme)	-	-	-	-	-	-	-

**2016**									
Adalimumab	-	Amjevita (Amgen)	-	-	-	Adfrar (Torrent Pharmaceutical)	-	-	-
Infliximab	Flixabi (Samsung Bioepis)	Inflectra (Pfizer)	-	-	-	-	-	-	-
Bevacizumab	-	-	Mvasi Amgen Canada INC	-	-	Bevacirel (Reliance Life Sciences)	-	-	-
	-	-	-	-	-	Cizumab (Hetero)	-	-	-
Etanercept	Benepali (Samsung Bioepis)	-	Brenzys (Samsung Bioepis)	-	-	-	-	-	-
	-	-	Erelzi (Sandoz)	-	-	-	-	-	-
Trastuzumab	-	-	-	-	HERtiCAD (Biocon)	-	-	-	-
**2015**									
Bevacizumab	-	-	-	-	Bevacizumab (Biocon)	-	-	-	-
Rituximab	-	-	-	-	-	Maball (Hetero Group)	-	-	-
	-	-	-	-	-	RituxiRel (Reliance life science)	-	-	-
Ranibizumab	-	-	-	-	-	Razumab (Intas Pharmaceutical)	-	-	-

Infliximab	-	-	Renflexis (Samsung Bioepis Co, Ltd)	Remsima (Celltrion)	Remsima (Celltrion)	-	-	-	-
Etanercept	-	-	-	-	-	Intacept (Intas Pharmaceutiacals)	-	-	-
**2014**									
Infliximab	-	-	Inflectra (Hospira)	-	-	Infimab (Epirus Biopharmaceuticals)	-	Remsima (Celltrion)	-
	-	-	Remsima (Celltrion)	-	-	-	-	-	-
Rituximab	-	-	-	-	MabThera /Rituxan (BIOCAD)	-	-	-	-
Adalimumab	-	-	-	-	-	Exemptia (Zydus Cadila)	-	-	-
**2013**									
Infliximab	Remsima (Celltrion Helathcare Hungary Kft)	-		-	-	-	-	-	-
	Inflectra (infliximab) Pfizer Europe MA EEIG	-	-	-	-	-	-	-	-
Trastuzumab Emtansine	-	-	Cadcyla (Hoffmann La Roche Ltd)	-	-	-	-	-	-

Abciximab	-	-	-	-	-	AbcixiRel (Reliance life science)	-	-	-
Trastuzumab	-	-	-	-	-	CanMab (Biocon)	-	-	-
Rituximab	-	-	-	-	-	Rituximab (Zenotech Lab)	-	-	-
	-	-	-	-	-	MabTas (Intas Pharmaceuticals)	-	-	-

MA- marketing authorization
**Halimatoz is approved for all indications as prescribed for Hefiya and additionally approved for rheumatoid arthritis.*
*** Rixathon is approved for all indications as prescribed for Riximyo and additionally approved for chronic lymphocytic leukemia.*
**** Truxima is approved for all indications as prescribed for Rituzena and additionally approved for chronic severe rheumatoid arthritis.*
***** Amgevita is approved for all indications as prescribed for Solymbix and additionally approved for chronic particular juvenile idiopathic arthritis.*

Table 2: List of agencies with reference guidelines

**Country**	**Agency name**	**Reference guidelines**
Europe	EMA(CHMP)	Guideline on development, production, characterisation and specification for monoclonal antibodies and related products EMA/CHMP/BWP/532517/2008, 2016.
		Guideline on similar biological medicinal products CHMP/437/04 Rev 1, 2014.
		Guideline on similar biological medicinal products containing biotechnology-derived proteins as active substance: non-clinical and clinical issues EMEA/CHMP/BMWP/42832/2005 Rev1, 2014.
		Guideline on process validation for the manufacture of biotechnology-derived active substances and data to be provided in the regulatory submission EMA/CHMP/BWP/187338/2014
		Guideline on similar biological medicinal products containing biotechnology-derived proteins as active substance: quality issues (revision 1) EMA/CHMP/BWP/247713/2012.
		Guideline on similar biological medicinal products containing monoclonal antibodies – non-clinical and clinical issues EMA/CHMP/BMWP/403543/2010, 2012.
		Guideline on the clinical investigation of the pharmacokinetics of therapeutic proteins CHMP/EWP/89249/2004, 2007.
		Development pharmaceutics for biotechnological and biological products EMA/CHMP/BMWP/403543/2010, 1999.
WHO	WHO Expert Committee on Biological Standardization	Guidelines on evaluation of monoclonal antibodies as similar biotherapeutic products (SBPs) WHO TRS No. 1004, 2017 Annex 2, Sixty-seventh report.
		WHO questions and answers similar biotherapeutic products WHO/SBP_Q&A/DRAFT/DEC 2017.
		Guidelines on evaluation of similar biotherapeutic products (SBPs) WHO TRS No. 977,

		2013 Annex 2, Sixtieth report.<br><br>Guideline for assuring the quality of monoclonal antibodies for use in humans WHO TRS No, 822, 1992
USA	USFDA(CBER)	Biosimilars: additional questions and answers regarding implementation of the biologics price competition and innovation act of 2009, 2018.<br><br>Scientific considerations in demonstrating interchangeability with a reference product guidance for industry, 2017.<br><br>Clinical pharmacology data to support a demonstration of biosimilarity to reference product guidance for industry, 2016.<br><br>Quality considerations in demonstrating biosimilarity of a therapeutic protein product to reference product guidance for industry, 2015 biosimilarity.<br><br>Scientific considerations in demonstrating biosimilarity to a reference product guidance for industry, 2015<br><br>Formal meetings between the FDA and sponsors or applicants of BsUFA products, Guidance for Industry, Draft Guidance 2018<br><br>Formal meetings between the FDA and biosimilar biological product sponsors or applicants 2015<br><br>Points to consider in the manufacture and testing of monoclonal antibody products for human use docket no. 94D-0259, 1997.
Canada	Health Canada/BGTD	Guidance document: information and submission requirements for biosimilar biologic drugs, 2016.<br><br>Fact sheet: biosimilars, 2017.<br><br>Guidance document: conduct and analysis of comparative bioavailability studies, file

		number: 12-105972-31, 2012.
Brazil	ANVISA	Provides on the registration of new biological products and biological products, giving other provisions RESOLUTION - RDC No. 55, December 16, 2010.
Russia	Russian federation	Registration dossier for finished medical product, Russian federal law no. 61-FZ.
India	CDSCO	Guideline on similar biologics: regulatory requirements for marketing authorization in India, 2016.
		Guideline on similar biologics: regulatory requirements for marketing authorization in India, 2012.
China	CFDA	Appendix Technical Guidelines for R&D and Evaluation of biosimilar (Trial)
South Africa	SAHPRA	Biosimilar medicines quality, non-clinical and clinical requirements 2.30_Biosimilars_Aug14_v3, 2014.
Turkey	TMMDA	Draft guideline on biosimilar medicinal products, 2015.
Mexico	COFEPRIS	Official mexican standard NOM-257-SSA1-2014, biotechnological medications, 2014.
ICH member countries	ICH	Development and manufacture of drug substances (chemical entities and biotechnological/biological entities) Q11, 2012.
		Pharmacovigilance planning E2E, 2004.
		Comparability of biotechnological/biological products subject to changes in their manufacturing process Q5E, 2004.
		Specifications: Test procedures and acceptance criteria for new drug substances and new drug products: chemical substances Q6A, 1999.
		Specifications: test procedures and acceptance criteria for biotechnological/biological products Q6B, 1999.

Viral safety evaluation of biotechnology products derived from cell lines of human or animal origin Q5A (R1), Version4 1999.

Derivation and characterisation of cell substrates used for production of biotechnological/biological products Q5D, 1997.

Quality of biotechnological products: analysis of the expression construct in cells used for production of r-DNA derived protein products Q5B, 1995.

Quality of biotechnological products: stability testing of biotechnological/biological products Q5C, 1995.

Table 3: Choice of reference product and related requirements

**Reference product selection**	**EMA**	**WHO**	**USFDA**	**HC/BGTD**
Selection of Reference product	Must be approved in EEA as per Article 8 of 2001/83/EC, as amended	Approved with full registration dossier regarding quality, efficacy, safety	FDA licensed single reference product	Approved in Canada
Non-authorized Reference product usage	Approved by ICH countries, can be used in certain non-/clinical, need to prove sameness between non-/ EEA RBP	Commercially available in well-established regulatory agency's market	Can be used for *in vivo* and clinical studies, bridging data with US reference product, prior consultation with FDA	Can be used from ICH adopting countries and Canada equivalent standards for comparability, evaluation and post-marketing surveillance
Bridging	to be provided in case of using non-EEA product	n/d	Non-US license product can be used for animal and clinical studies, must use US license product for analytical studies, PK and PD study one each, adequate bridging data justifying clinical trial design supporting conditions of use and patient population, relationship between non-licensed, component manufacturers if any and BLA license holder, relevance of GMP issuing authority for non-licensed product	Essential for analytical and PK/PD comparison for all product
Identity of Reference product	n/d	Should be identifiable	n/d	n/d

Sameness of Reference product	Non EEA product can be used together EEA product for defining QTPP during development, analytical and clinical PK/PD studies between non-EEA, EEA and proposed biosimilar, Prior consultation with agency	The same RBP should be used throughout the comparative quality, nonclinical, and clinical studies	n/d	Possible to use more than one reference biologic drug in clinical studies, sourced from Non-Canadian markets

Q,S,E: Quality, safety, efficacy
n/d: Not defined

Table 4: Choice of reference product & related requirement for BRICS-TM

**Reference product selection**	**ANVISA**	**Russian federation**	**CDSCO**	**CFDA**	**SAHPRA**	**TMMDA**	**COFEPRIS**
Selection of Reference product	Approved based on full registration dossier with ANVISA Brazil	Biosimilar products	Should be licensed in India or ICH countries, Innovator product, approved by full dossier including quality, safety and efficacy	China approved product is mandatory for clinical comparison study	Registered with MCC based on complete quality, safety and efficacy data and innovator product	Reference medicinal product must be authorized with complete dossier by competence authorities	Should have valid registration issued by COFEPRIS, commercially available in Mexico
Non Reference product	Non Brazil reference product from countries having similarity with ANVISA and access to full dossier.	Not defined	Non ICH reference product sourcing not defined	Not defined	Sourced from MCC aligning countries	Not defined	Biosimilar can be used as reference product subject to biosimilarity has been demonstrated
Bridging	Not defined	Not defined	Not defined	Not defined	Not defined	No need	Not defined
Identity of Reference product	Not defined	Not defined	Not defined	Not defined	Not defined	Should be identifiable (brand name, pharmaceutical form, formulation, manufacturing & expiration date)	Not defined

Sameness of Reference product	Same biological product is used throughout the comparability exercise	Not defined	Same reference product throughout comparibility study	Expected to use same source of origin throughout comparibility study	Not defined	Single reference product throughout comparability study of Q/S/E	Not defined

Table 5: Differences in biosimilarity criteria across the well-established and emerging agencies

	**EMA**	**WHO**	**USFDA**	**BGTD**	**ANVISA**	**Russian federation**	**CDSCO**	**CFDA**	**SAHPRA**	**TMMDA**	**COFEPRIS**
Posology	Same as RBP	n/d	Same as RBP	n/d	n/d	n/d	Same as RBP	n/d	n/d	Same as RBP	n/d
Route of administration	Same as RBP	Same as RBP	Same as RBP	Same as RBP	n/d	n/d	Same as RBP	n/d	n/d	Same as RBP	n/d
Strength, Pharmaceutical form, Formulation	Variation acceptable with justification, no compromise with safety. Molecularly and biologically same active ingredient	Change acceptable without impact on Q,S,E	Strength can be different, Pharmaceutical form must be same as reference product, Formulation can be different, Inactive part can be different, acceptable with clinically no meaningful difference	Strength and form should be same as RBP, not specified for formulation	n/d	n/d	Same strength, other criteria n/d	n/d	n/d	Variation acceptable with justification, no compromise with safety. Molecularly and biologically same active ingredient	n/d
Improved efficacy	Not suitable	Not suitable	n/d	n/d	n/d	n/d	n/d	n/d	n/d	Not suitable	n/d

Improved safety	Low impurity profile or less immunogenicity, acceptable	Low impurity acceptable	n/d	Highly similar or same level (% of impurities)	n/d	n/d	n/d	n/d	n/d	Low impurity profile/less immunogenicity, acceptable	n/d
Extrapolation of indications	Acceptable with justification	Acceptable under certain circumstances	Acceptable with scientific justification, recommended to perform comparibility studies in sensitive condition and studied under post-marketing surveillance	Acceptable with justification	possible if developed by comparability route and not by individual development	n/d	Acceptable subject to clinical safety and efficacy in one indication, MOA same for all indications and other conditions are met	Acceptable with comparative clinical study in particular indication, MOA same for all indications	Possible based on biosimilarity in particular indication, main clinical trial with non-inferiority design, justification based on published and pharmacopoeial proof	Acceptable with justification	No extrapolations between indications
Biosimilarity post approval	No need to prove biosimilarity	n/d	n/d	n/d	n/d	n/d	n/d	n/d	n/d	No need to prove	n/d
Interchangeabilit	To be regulated by	To be defined	Interchangeability approved	Interchangeability	n/d	n/d	n/d	n/d	Not interchan	Substitution is not	n/d

y, Switching and Substitution	member states and not EMA	by NRA	subject to clinical result is same as reference product in any given patient and proved for all licensed conditions of use	authorized by provinces and territory					geable, non-switchable	allowed, and interchangeability is possible with practitioner's decision	
Pediatric research	Pediatric Investigational plan and/or pediatric waiver/deferral submission not applicable for biosimilar	n/d	Extrapolation of efficacy in pediatric population is permitted under PREA subject to conditions are met	n/d	n/d	n/d	n/d	n/d	n/d	n/d	n/d

Table 6: Comparative evaluation of mAb physico-chemical characterization

	EMA	WHO
**mAb structure**		
Primary and higher order structure	Class and subclass determination, kappa and/or lambda chain and primary structure to be characterized	To be characterized but not specified requirement
Amino acid	Amino acid sequencing and variability of N- and C-terminal to be confirmed	n/d
Groups and bridges	Free sulphydryl groups and disulfide bridges to be determined, integrity and mismatch of bridge to be analyzed	n/d
Carbohydrate	Carbohydrate content and structure, oligosaccharide pattern to be confirmed	Carbohydrate structures to be defined
Glycosylation	Presence or absence of additional glycosylation site(s) on Fc region to be confirmed, glycosylation site(s) with occupancy and additional glycosylation site(s) in the heavy chains to be analyzed	Evaluation of glycosylation pattern including site occupancy
Glycan/ Isoforms	Glycan structure to be characterized for degree of mannosylation, galactosylation, fucosylation and sialylation with distribution of main glycan structures to be determined	Comprehensive evaluation including number or type of glycans and qualitative identification incase glycan non-existent in human, analysis of glycan attached to Fc- region
**Immunological properties**		
Antigen binding assay	Antigens binding assay at defined regions including affinity, avidity and immunoreactivity as feasible	Binding assays to be performed but not defined in detailed
Cytotoxicity evaluation	For unintended target tissue to be evaluated	n/d
Cross-reactivity	To be determined	n/d
CDR	To be identified	n/d
Epitope	Characterization, biochemical identification and determination of epitope including bearing molecules	n/d

Complementary ability evaluation	Evaluation of binding and activation and/or effector functions	n/d
**Biological activity**		
In vitro/vivo assay	Assessment of biological activity by *in vitro*/*vivo* assay to be justified if required	Indicated as appropriate assay to be done but not defined
Product effector functions	ADCC analysis, cytotoxic properties (e.g. apoptosis), complement binding ability, Fc- gamma receptor binding activity and neonatal receptor binding ability performed incase mechanism of action impact S &E	ADCC, binding ability to Fc$^{\gamma}$ and neonatal Fc receptors to be performed, not specified if MoA doesn't impact S&E, complement C1q test required, Fc- and Fab- related function to be evaluated
**Purity, impurity and contaminants**		
Purity	By orthogonal methods	Methods not defined
Structural heterogeneity	Qualitative and quantitative analysis to be investigated	To be investigated, identified and quantified
Multimers, aggregates and particulates	To be characterized and monitored	n/d
Impurity profile and Process-related impurities	Qualitative and/or quantitative evaluation	System- specific process impurities to be considered.
Contaminants	Controlled/additional testing to be done	n/d
**Cell lines**		
Cell lines/ Expression system	Sufficient information to be provided but detailed procedures not required	Different cell lines allowed, advised to use RBP similar system
Immortalization approach	To be justified	n/d
Hybridoma cell lines	Origin and characteristics of parental cell to be documented	n/d
**Quantity**		
Basis for quantity determination	Biological assay if correlated	Biological activity and expression system
**Specifications**		

Specification determination	Based on number and age of lots, time of testing and types of quality attributes	Based upon the manufacturer's experience with SBP and experimental results of SBP and RBP
Tests selection	As per ICH Q6B, product specific for drug substance and drug product	Pharmacopoeial monograph plus additional test
Acceptance criteria	Based on lots used in different studies (manufacturing consistency, clinical and non-clinical studies, stability studies and relevant development data)	Based on sufficient lots, should not be wider than variability range of RBP during shelf life
Validated methods for characterization	To be submitted in dossier	Scientifically sound and qualified but not necessarily validated
Analytical methods for lot release	To be validated	To be validated
Reference materials and Standard	Ph Eur. and WHO	WHO
Accelerated stability data	Should be part of characterization study	Accelerated degradation and stress studies (non-comparable), Comparative head-to-head accelerated stabilities studies between SBP and RBP, drug product and drug substance stability in intended and representing container closure system simultaneously
Experimental stability data	Formulation data with different quantities of excipient	n/d
In-process stability data	To be performed in-case of lyophylization	n/d
Routine stability study	Based on ICH Q5C	Based on NRA

n/d: Not applicable
S&E: Safety and efficacy

73

Table 7: Comparative quality (characterization) attributes for BRICS-TM markets

Characterization	ANVISA	Russian federation	CDSCO	CFDA	SAHPRA	TMMDA	COFEPRIS
**mAb structure**							
Primary and higher order structure	To be characterized	n/d	To be characterized but not specified requirement	To be characterized	Class and subclass determination, kappa and/or lambda chain and primary structure to be characterized	To be characterized	To be characterized but not specified requirement
Amino acid	n/d	n/d	n/d	n/d	Amino acid sequencing and variability of N- and C- terminal to be confirmed	n/d	n/d
Groups and bridges	n/d	n/d	n/d	To be characterized	Free sulphydryl groups and disulfide bridges to be determined, integrity and mismatch of bridge to be analyzed	To be justified, if difference detected with reference product	n/d
Carbohydrate	n/d	n/d	Carbohydrate structures to be defined	n/d	Carbohydrate content and structure, oligosaccharide pattern to be confirmed	n/d	Carbohydrate structures to be defined

Glycosylation	n/d	n/d	Evaluation of glycosylation pattern including site occupancy	To be characterized	Presence or absence of additional glycosylation site(s) on Fc region to be confirmed, glycosylation site(s) with occupancy and additional glycosylation site(s) in the heavy chains to be analyzed	n/d	Evaluation of glycosylation pattern including site occupancy
Glycan/ Isoforms	n/d	n/d	Comprehensive evaluation including number or type of glycans and qualitative identification incase glycan non-existent in human, analysis of glycan attached to Fc-region	n/d	Glycan structure to be characterized for degree of mannosylation, galactosylation, fucosylation and sialylation with distribution of main glycan structures to be determined	n/d	Comprehensive evaluation including number or type of glycans and qualitative identification incase glycan non-existent in human, analysis of glycan attached to Fc- region
**Immunological properties**							
Antigen binding assay	n/d	n/d	n/d	Fab & Fc region	n/d	Fab & Fc region	n/d
Cytotoxicity evaluation	n/d	n/d	n/d	CDC & ADCC activity	n/d	n/d	n/d

Cross-reactivity	n/d	n/d	n/d	n/d	n/d	n/d	n/d
CDR	n/d	n/d	n/d	n/d	n/d	n/d	n/d
Epitope	n/d	n/d	n/d	n/d	n/d	n/d	n/d
Complementary ability evaluation	n/d	n/d	n/d	FcRn & Fc & C1q receptor affinity	n/d	FcRn & Fc & C1q receptor affinity	n/d
**Biological assays**							
In vitro/vivo assay	Required but no detailed guideline	n/d	Required but no detailed guideline	Bioactivity test	Required but no detailed guideline	Binding, enzymatic, cell-based, functional assays	n/d
Approach	n/d	n/d	n/d	n/d	n/d	Complementary or orthogonal approaches	n/d
Product effector functions	n/d	n/d	n/d	n/d	n/d	n/d	n/d
**Purity, impurity and contaminants**							
Purity	n/d	n/d	Orthogonal method remains unspecified	Hydrophobicity, charge & molecular size variant, post translation modification	n/d	In line to EMA	n/d
Structural heterogeneity	n/d	n/d	Orthogonal method remains unspecified	n/d	n/d	n/d	n/d
Multimers, aggregates and particulates	n/d	n/d	Should be evaluate	n/d	aggregates formation test	n/d	n/d

Impurity profile and Process-related impurities	To be performed.	n/d	Process-related impurities should be evaluate	Required but no detailed guideline	Required but no detailed guideline	Process-related & product-related impurities should be evaluate	n/d
Contaminants	To be performed.	n/d	Orthogonal method remains unspecified	n/d	n/d	In line to EMA	n/d
**Cell lines**							
Cell lines/ Expression system	n/d	n/d	n/d	n/d	n/d	n/d	n/d
Immortalization approach	n/d	n/d	n/d	n/d	n/d	n/d	n/d
Hybridoma cell lines	n/d	n/d	n/d	n/d	n/d	n/d	n/d
**Quantity**							
Basis for quantity determination	n/d	n/d	n/d	n/d	n/d	Should be describe	n/d
**Specifications**							
Specification determination	n/d	n/d	n/d	consistent with reference product	n/d	n/d	n/d
Tests selection	n/d	n/d	n/d	Sensitive & advanced	n/d	n/d	n/d
Acceptance criteria	n/d	n/d	n/d	n/d	n/d	n/d	n/d
Validated methods for characterization	n/d	n/d	Qualified assay	n/d	Qualified assay	n/d	n/d

Analytical methods for lot release	n/d	n/d	n/d	Advanced method to be used	n/d	n/d	n/d
Reference materials and Standard	n/d	n/d	n/d	n/d	n/d	n/d	n/d
Accelerated stability data	n/d	n/d	n/d	Required but no detailed guideline	n/d	n/d	n/d
Experimental stability data	n/d	n/d	n/d	n/d	n/d	n/d	n/d
In-process stability data	n/d	n/d	n/d	n/d	n/d	n/d	n/d
Routine stability study	n/d	n/d	n/d	Required but no detailed guideline	n/d	Claimed shelf life obtained from full stability data	n/d

n/d: Not defined

Table 8: Comparative clinical attributes across BRICS-TM markets

	ANVISA	Russian federation	CDSCO	CFDA	SAHPRA	TMMDA	COFEPRIS
**Pharmacokinetics**							
Dose	n/d	n/a	Prefer lowest therapeutic dose. Higher dose for mAb clearance characteristics	n/d	Lowest therapeutic dose	Lowest therapeutic dose	n/d
ROA	n/d	n/a	Subcutaneous routes	n/d	Subcutaneous routes	Subcutaneous routes	n/d
Sampling	n/d	n/a	Single dose: Till last quantifiable concentration; Multi dose: First dose & steady state	n/d	Single-dose: First & last administration; Multiple-dose: Steady state	Single-dose: First & last administration; Multiple-dose: Steady state	n/d
Design	n/d	n/a	Single-dose cross-over for late elimination phase; Parallel group for long half-life	single/multiple dose	Single-dose cross-over for late elimination phase; Parallel group for long half-life	Single-dose cross-over for late elimination phase; Parallel group for long half-life	n/d
Primary parameter	n/d	n/a	n/d	n/d	Single dose: $AUC_{(0\text{-}inf)}$ Multiple dose: C_{max} & C_{trough}	Single dose: $AUC_{(0\text{-}inf)}$ Multiple dose: C_{max} & C_{trough}	n/d

Secondary parameter	n/d	n/d	n/d	n/d	Single dose: C_{max}, T_{max}, V_{ss}, $t_{1/2}$; Multiple dose: $AUC_{(0-t)}$, steady state AUC	Single dose: C_{max}, T_{max}, V_{ss}, $t_{1/2}$; Multiple dose: $AUC_{(0-t)}$, steady state AUC	n/d
Acceptable range	n/d	n/a	Clinically justified	n/d	Clinical judgment	Clinical judgment	n/d
**Pharmacodynamics**							
Combined PKPD	Possible if PD marker available	n/a	Comparative, parallel/cross-over, healthy volunteers/patient if PD marker available	Possible if PD marker available	Possible if PD marker available	n/d	n/d
Fingerprinting approach	n/d	n/a	n/d	n/d	n/d	n/d	n/d
**Clinical efficacy**							
Study type	Required but no detailed guideline	n/a	randomized, parallel group, blinded	parallel design, random, double-blind	n/d	n/d	n/d
Population	n/d	n/a	n/d	Patient for approved therapeutic indication	n/d	n/d	n/d
Design	n/d	n/a	equivalence, non-inferiority or comparability phase III clinical trial	equivalent efficacy design trial	clinical comparability trial	n/d	n/d
Endpoints	n/d	n/a	n/d	secondary endpoints	n/d	n/d	n/d

Comparability margin	n/d	n/a	n/d	Justified by considering assay sensitivity	n/d	n/d	n/d
Pediatric population	n/d	n/a	n/d	n/d	n/d	n/d	n/d
**Clinical Safety**							
Immunogenicity	n/d	n/a	Obtained in PKPD studies	Required but no detailed guideline	Required but no detailed guideline	n/d	n/d
Comparative safety data	Required but no detailed guideline	n/a	Obtained in PKPD studies if phase III trial is waived	Adverse reaction comparison to be done with reference drug	In line with EMA	n/d	n/d
Follow-up duration	n/d	n/a	n/d	n/d	In line with EMA	n/d	n/d

n/d: Not defined
n/a: Not available
